# PCYT1A deficiency disturbs fatty acid metabolism and induces ferroptosis in the mouse retina

**DOI:** 10.1186/s12915-024-01932-y

**Published:** 2024-06-10

**Authors:** Kaifang Wang, Huijuan Xu, Rong Zou, Guangqun Zeng, Ye Yuan, Xianjun Zhu, Xiaohui Zhao, Jie Li, Lin Zhang

**Affiliations:** 1 The Sichuan Provincial Key Laboratory for Human Disease Gene Study, Center for Medical Genetics, Sichuan Provincial People’s Hospital, School of Medicine, University of Electronic Science and Technology of China, Chengdu, 610072 Sichuan China; 2 Qinghai Provincial Key Laboratory of Tibetan Medicine Research, Northwest Institute of Plateau Biology, Chinese Academy of Sciences, Xining, 810008 Qinghai China; 3 The People’s Hospital of Pengzhou, Chengdu, 611930 Sichuan China; 4 Medical Center Hospital of Qionglai City, Chengdu, 611530 Sichuan China; 5 Research Unit for Blindness Prevention of Chinese Academy of Medical Sciences (2019RU026), Sichuan Academy of Medical Sciences and Sichuan Provincial People’s Hospital, Chengdu, 610072 Sichuan China; 6 Department of Ophthalmology, Sichuan Provincial People’s Hospital, School of Medicine, University of Electronic Science and Technology of China, Chengdu, 610072 Sichuan China

**Keywords:** IRDs, PCYT1A, Fatty acid metabolism, Ferroptosis

## Abstract

**Background:**

Inherited retinal dystrophies (IRDs) are a group of debilitating visual disorders characterized by the progressive degeneration of photoreceptors, which ultimately lead to blindness. Among the causes of this condition, mutations in the *PCYT1A* gene, which encodes the rate-limiting enzyme responsible for phosphatidylcholine (PC) de novo synthesis via the Kennedy pathway, have been identified. However, the precise mechanisms underlying the association between *PCYT1A* mutations and IRDs remain unclear. To address this knowledge gap, we focused on elucidating the functions of *PCYT1A* in the retina.

**Results:**

We found that PCYT1A is highly expressed in Müller glial (MG) cells in the inner nuclear layer (INL) of the retina. Subsequently, we generated a retina-specific knockout mouse model in which the *Pcyt1a* gene was targeted (Pcyt1a-RKO or RKO mice) to investigate the molecular mechanisms underlying IRDs caused by *PCYT1A* mutations. Our findings revealed that the deletion of* Pcyt1a* resulted in retinal degenerative phenotypes, including reduced scotopic electroretinogram (ERG) responses and progressive degeneration of photoreceptor cells, accompanied by loss of cells in the INL. Furthermore, through proteomic and bioinformatic analyses, we identified dysregulated retinal fatty acid metabolism and activation of the ferroptosis signalling pathway in RKO mice. Importantly, we found that PCYT1A deficiency did not lead to an overall reduction in PC synthesis within the retina. Instead, this deficiency appeared to disrupt free fatty acid metabolism and ultimately trigger ferroptosis.

**Conclusions:**

This study reveals a novel mechanism by which mutations in PCYT1A contribute to the development of IRDs, shedding light on the interplay between fatty acid metabolism and retinal degenerative diseases, and provides new insights into the treatment of IRDs.

**Supplementary Information:**

The online version contains supplementary material available at 10.1186/s12915-024-01932-y.

## Background

**Fig. 1:**
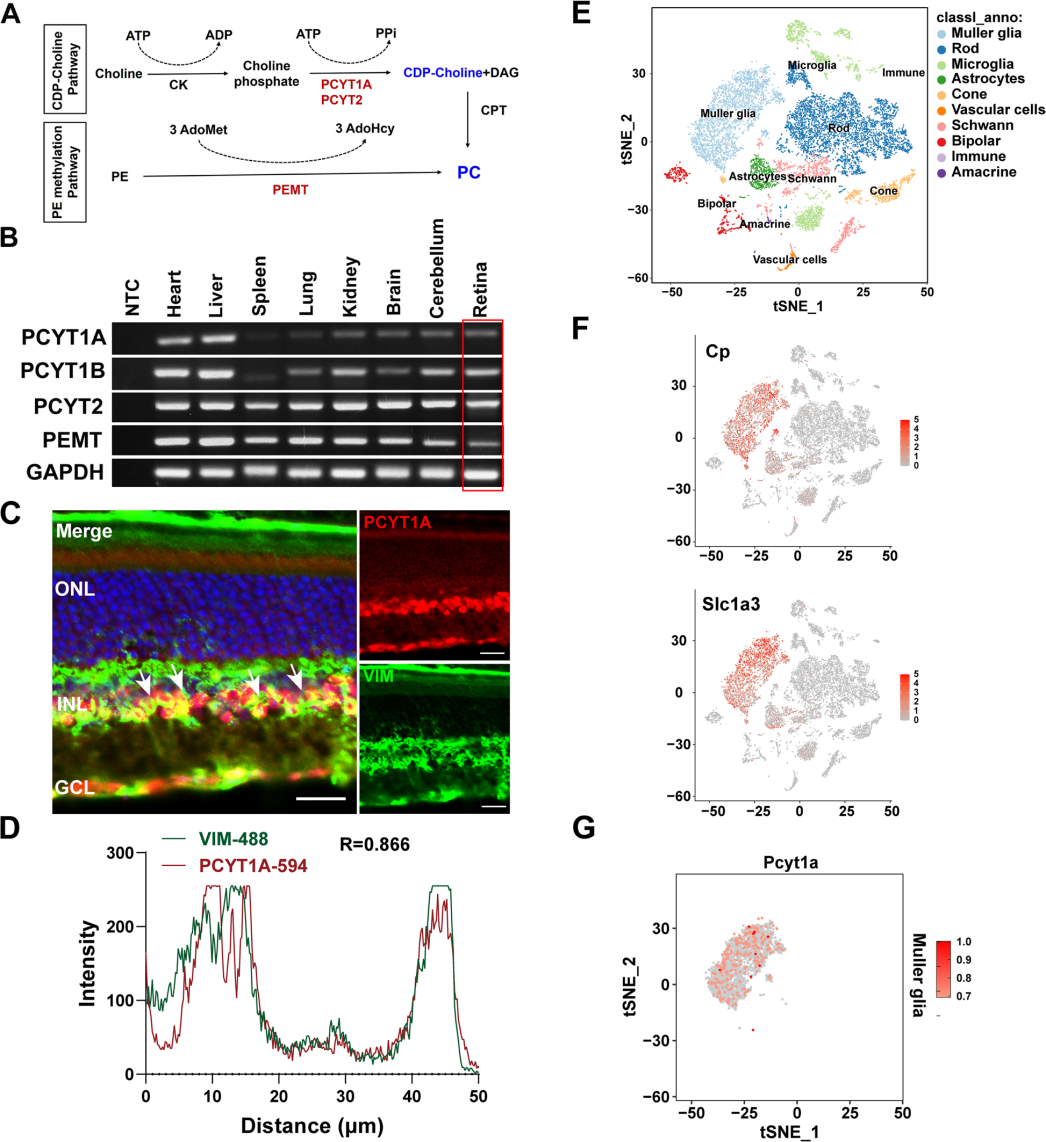
Expression of PCYT1A in the mouse retina. **A** Figure indicating the key processes and enzymes in the two pathways for PC synthesis: the CDP-choline (Kennedy pathway) and PE methylation pathways. **B** RT-PCR experiments showing the expression of key enzymes related to PC synthesis in various tissues. **C** Immunofluorescence staining of retinas from 2-month-old mice suggested that the PCYT1A protein was mainly expressed in the retinal INL. Scale bar, 20 μm. ONL, outer nuclear layer; INL, inner nuclear layer; GCL, ganglion cell layer. **D** Colocalization analysis of PCYT1A and VIM. **E** The t-SNE plot of single-cell sequencing of eye tissue from 3-month-old mice revealed 10 distinct cell clusters in the retina. **F** Expression levels of selected MG marker genes (Cp and Slc1a3). **G** t-SNE plot of *Pcyt1a* gene expression in the MG cell cluster

Cell and organelle membranes are composed of a complex phospholipid (PL) bilayer that determines their shape, size, and function [1]. Phosphatidylcholine (PC) is the most abundant PL in eukaryotic membranes, accounting for approximately 30–60% of the total PL amount [2]. In addition to being a structural component of biomembranes, PC is also critically involved in cell proliferation, differentiation, hormone and lipoprotein secretion, and lung surfactant [3–6]. There are two pathways for the de novo biosynthesis of PC, namely, the Kennedy and phosphatidylethanolamine (PE) methyltransferase pathways [7]. The Kennedy pathway, also known as the CDP-choline pathway, is the major route for PC synthesis in most mammalian tissues [8]. In this pathway, cells synthesize PC in three consecutive steps (Fig. 1A): choline kinase phosphorylates choline, and then, phosphocholine cytidylyltransferase generates the high-energy donor CDP-choline in the rate-limiting step of the pathway [9]. The rate-limiting enzyme for this pathway is CTP: phosphocholine cytidylyltransferase (CCT), which catalyzes the synthesis of CDP-choline [10]. In mammals, two genes encode CCT, *PCYT1A* and *PCYT1B*, which encode CCTα and CCTβ, respectively [11]. Of these two CCTs, CCTα has broader and greater tissue expression and is the predominant enzyme involved in PC synthesis [4, 12].

In addition to the important function of CCTα in PC synthesis, genetic studies revealed that loss-of-function mutations in the *PCYT1A* gene were associated with three diseases: lipodystrophy (c.838_840delCTC, p.E280del) [9], spondylometaphyseal dysplasia with cone-rod dystrophy (SMD-CRD) (c.296C-T transition, p.A99V; c.448C-G transversion, p.P150A) [13, 14], and isolated retinal dystrophy (c.897 + 1G4A, p.A93T, p.R283*) [15]. Both cone-rod dystrophy and isolated retinal dystrophy are inherited retinal dystrophies (IRDs). IRDs are a heterogeneous group of conditions characterized by the progressive degeneration of photoreceptors and/or retinal pigment epithelial cells [16, 17] and are the most frequent causes of visual impairment with a genetic origin, with a prevalence of 1:3000 to 1:2000 worldwide [18]. IRDs consist of no less than 20 clinical phenotypes and display notable genetic heterogeneity with more than 300 causative genes [19]. The heterogeneity of IRDs is reflected not only in the large number of causative genes but also in their diverse pathogenic mechanisms [20, 21]. Elucidating the pathogenic mechanisms of IRDs is crucial for the treatment of this disease.

Although *PCYT1A* has been proven to be a causative gene of IRDs, the functions of this gene in the retina and pathogenic mechanisms have not been fully elucidated. The fundamental function of CCTα has long been studied, and its in-depth functions in cellular biochemical processes have been revealed in recent years. Gabriela Andrejeva et al. discovered that the loss of CCTα activity resulted in compromised autophagosome formation and maintenance in autophagic cells, demonstrating the important function of de novo PC synthesis by CCTα in the autophagic process [22]. Several other groups focused on the interesting roles of CCTα in the biogenesis of lipid droplets (LDs) or nuclear lipid droplets (nLDs), demonstrating that CCTα may be regulated by diacylglycerol (DAG) to coordinate PC and triacylglycerol (TAG) synthesis when cells are exposed to excess exogenous fatty acids [10, 23], although the details of CCTα involvement in LD or nLD formation remain to be elucidated. In addition to these functions, CCTα also plays important roles in the formation of the nucleoplasmic reticulum [24], maintenance of the nuclear membrane structure [25], and the membrane stored curvature elastic stress response [2]. The diverse cellular functions of CCTα complicate its involvement in the pathogenic mechanisms of disease. Thus, we generated a retina-specific *Pcyt1a* knockout mouse model to investigate the molecular mechanism of IRDs caused by *PCYT1A* mutations in this study. We showed that PCYT1A deficiency caused retinal degenerative phenotypes, attenuated electroretinogram (ERG) responses, and promoted progressive degeneration of photoreceptor cells. Further mechanistic studies demonstrated that PCYT1A deletion did not cause abnormal overall PC synthesis in the retina but impaired fatty acid metabolism and eventually caused ferroptosis. Our study highlights the relationship between PCYT1A deletion and lipid metabolic homeostasis and ferroptosis and reveals a novel mechanism by which *PCYT1A* mutations cause IRDs, shedding light on the treatment of IRDs.

## Results

### Expression of key genes related to PC synthesis in the mouse retina

In this study, we first examined the expression of several key genes related to PC synthesis in different tissues, including the heart, liver, spleen, lung, kidney, brain, cerebellum, and retina. PCYT1A and its isoform PCYT1B are related to the Kennedy pathway, while PCYT2 and PEMT are key enzymes in the PE methyltransferase pathway (Fig. 1A). RT-PCR assays revealed that the mRNAs of these four genes were expressed in the retina (Fig. 1B), indicating that the two pathways for PC synthesis are active in the retina. These findings prompted us to explore the pathogenic mechanism of IRDs caused by *PCYT1A* mutations. Localization of PCYT1A in the retina was shown by immunohistochemical analysis of sections of mouse retinas with an anti-PCYT1A antibody. As shown in Fig. 1C, PCYT1A was mainly localized in the nucleus of the inner nuclear layer (INL) and retinal ganglion cell (RGC) layer, with less expression in the outer nuclear layer (ONL). Figure 1D shows that the Pearson correlation coefficient (*R* = 0.866) between PCYT1A and vimentin (VIM, an MG cell marker) was significant and positive. We analysed single-cell RNA-seq data from the eye tissues of 3-month-old wild-type mice [26, 27]. After filtering out low-quality cells using Seurat software (Additional file 1: Fig. S1A), we included a total of 14,357 cells for downstream analysis. Subsequently, we visualized single-cell clustering using the t-SNE dimensionality reduction approach and defined 10 cell clusters (Fig. 1E). The characteristic gene markers for each cell cluster are shown in Additional file 1: Fig. S1B. We focused on the expression pattern of the *Pcyt1a* gene across the cell clusters (Additional file 1: Fig. S1C) and found that *Pcyt1a* was predominantly expressed in MG cells (Fig. 1F, G). These results proved that PCYT1A is highly expressed in MG cells in the retina.

### Loss of Pcyt1a in the retina leads to retinal degeneration

To investigate the in vivo roles of *Pcyt1a* in the retina, we crossed *Pcyt1a* conditional knockout mice (named Pcyt1a^fl/+^) with Six3-Cre transgenic mice [28] to generate a retina-specific *Pcyt1a* knockout mouse model (Additional file 1: Fig. S2A). This transgenic line has been widely used by multiple groups investigating the function of genes of interest in the retina, the efficiency of which has been verified [29–32]. *Pcyt1a*^flox/flox^ mice were mated to *Six3-Cre* transgenic mice to generate *Pcyt1a*^RKO/RKO^ mice with alleles to inactivate *Pcyt1a* in the retina; these mice are referred to as RKO hereafter. Pcyt1a^fl/fl^ mice were used as Ctl mice for further experiments. PCR was used for genotyping the offspring (Additional file 1: Fig. S2B). Immunoblotting revealed that the protein expression level of PCYT1A in the RKO mice was significantly lower than that in the Ctl mice (only approximately 15% of that in the Ctl mice) (Additional file 1: Fig. S2C), demonstrating that the RKO mouse model was constructed successfully.

**Fig. 2:**
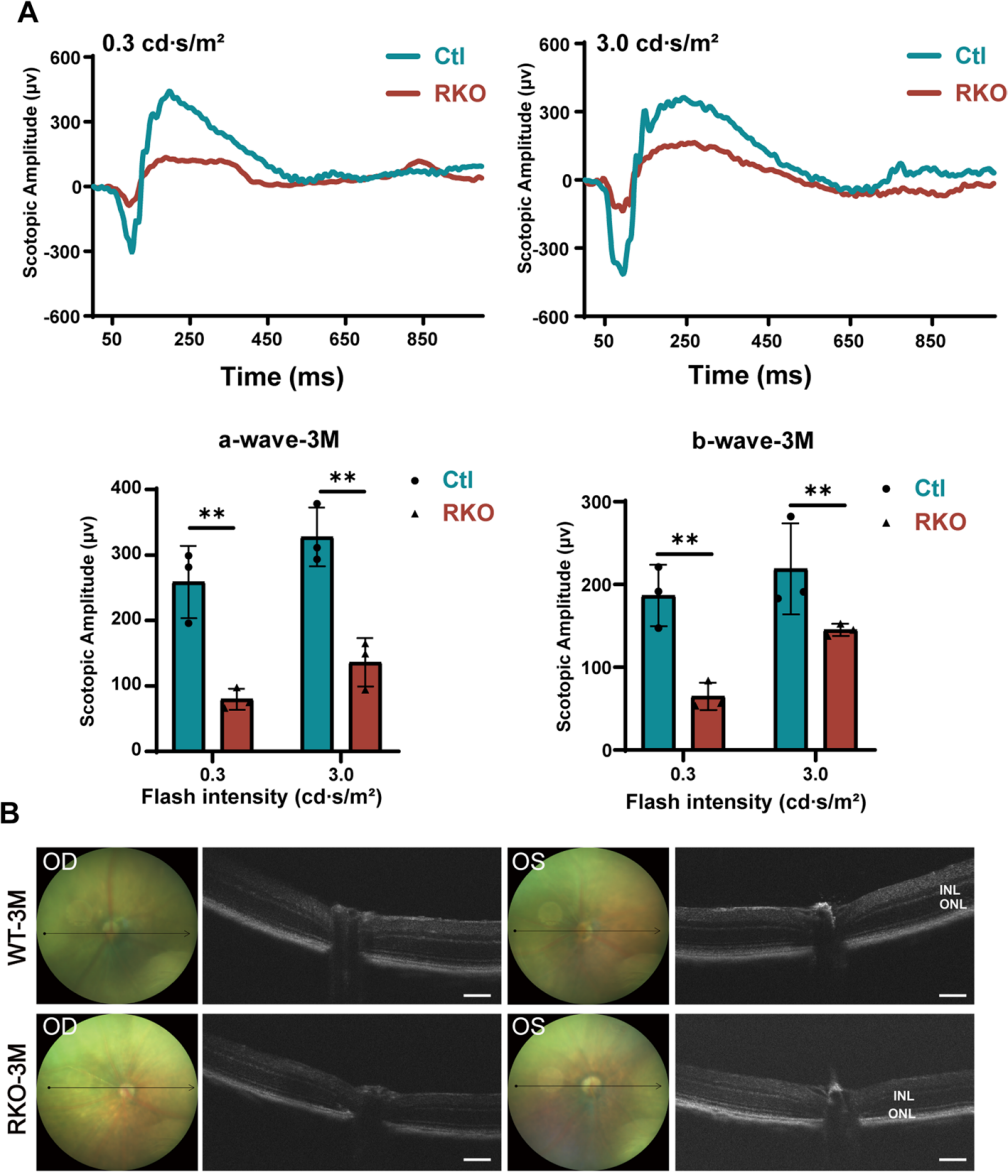
ERG and OCT were performed on 3-month-old RKO and Ctl mice. **A** ERG examination showed that RKO mice exhibited an attenuated ERG scotopic response at both 0.3 and 3 cd s/m^2^ flash intensities. ERG experiments were tested on 6 mice, *n* = 3 for Ctl mice, *n* = 3 for RKO mice. **P < 0.01 by multiple Student’s *t*-tests. **B** Representative fundus images and OCT scanning photos of Ctl and RKO mice. Scale bar, 100 μm. All data are shown as mean ± SD

**Fig. 3:**
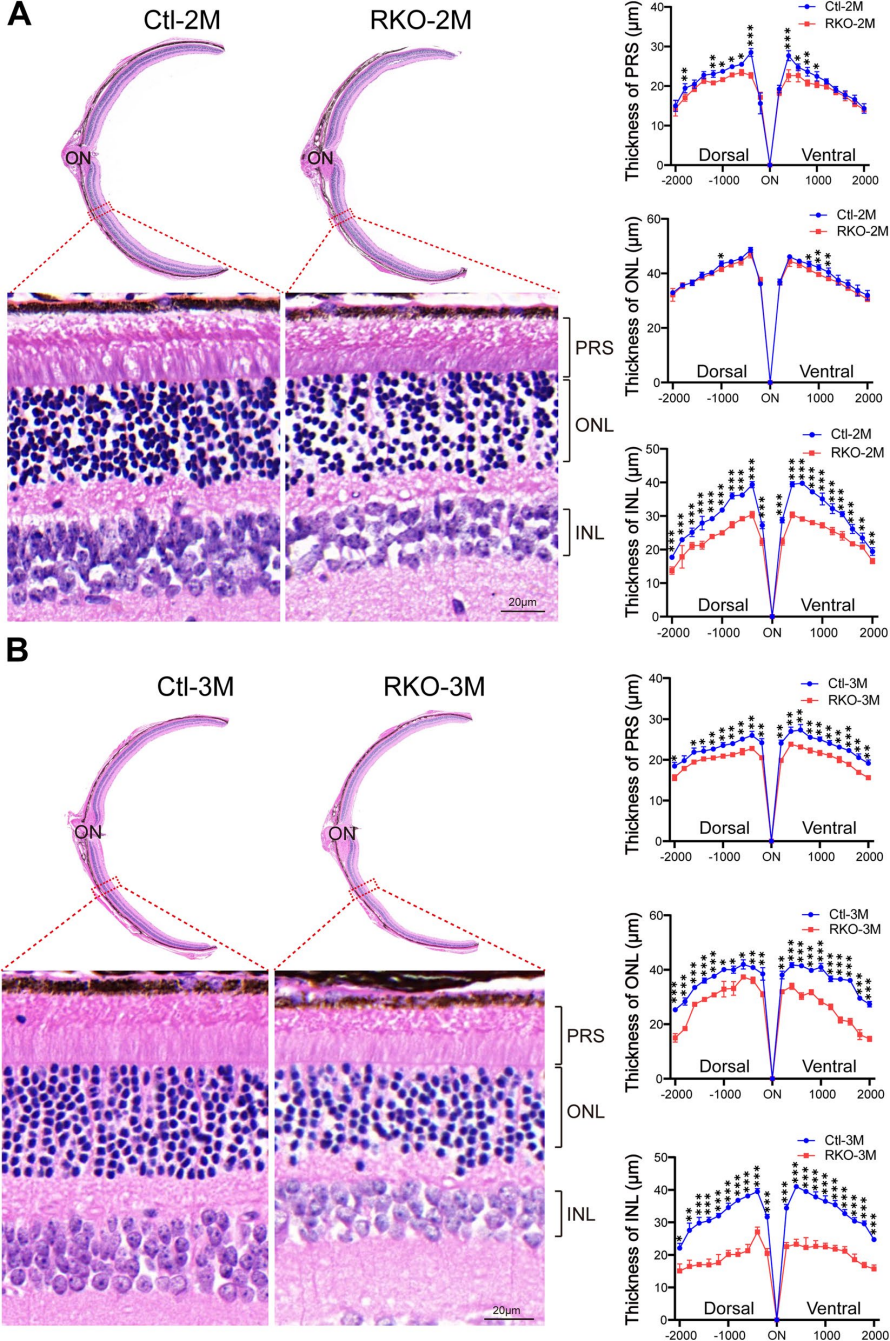
H&E staining revealed a progressive degenerative phenotype in RKO mice. H&E staining of paraffin sections of RKO and corresponding control retinas at the ages of 2 (**A**) and 3 (**B**) months. Scale bar, 20 μm. The right panel shows the quantification of the thickness of the PRS, ONL, and INL of 6 retinas from 3 Ctl mice and 6 retinas from 3 RKO mice at different ages. Multiple Student’s *t-*tests were used for statistical analysis. PRS, photoreceptor segment; ONL, outer nuclear layer; INL, inner nuclear layer. The thickness of the different layers of the retina was measured at intervals of 200 μm at the Dorsal side and Ventral side, using the optic centre of the retina as the starting point. *P < 0.05, **P < 0.01, ***P < 0.001. All data are shown as mean ± SD

To test the overall retinal function of the RKO mice, we performed an ERG on 3-month-old RKO mice and littermate controls. The RKO mice exhibited an attenuated scotopic response: both the a-wave and b-wave amplitudes were diminished at 0.3 and 3.0 cd s/m^2^ flash intensities (Fig. 2A), indicating impaired rod cell function. Then, we performed OCT analysis on mice of different ages. The RKO mice at 2 months of age showed slightly reduced retinal thickness (Additional file 1: Fig. S3), while retinal thickness was significantly reduced at 3 months of age (Fig. 2B). Further analysis revealed that retinal thinning in the RKO mice was mainly caused by a reduction in the thickness of the ONL and INL. We then carried out H&E staining of retinal sections from the RKO and Ctl mice at 2, 3, and 10 months of age to evaluate pathological changes in the RKO mice. Only the retinas of the 2-month-old RKO mice showed a slight reduction in thickness in the INL layer (Fig. 3A). Consistent with the OCT findings, the 3-month-old RKO mice exhibited the following retinal degenerative phenotypes: reduced photoreceptor segment (PRS) and reduced ONL and INL thickness (Fig. 3B). In the 10-month-old RKO mice, the retinal degenerative phenotype became more pronounced (Additional file 1: Fig. S4). These findings demonstrated that the RKO mice exhibited progressive retinal degeneration and that the degeneration appeared to initiate in the INL.

**Fig. 4:**
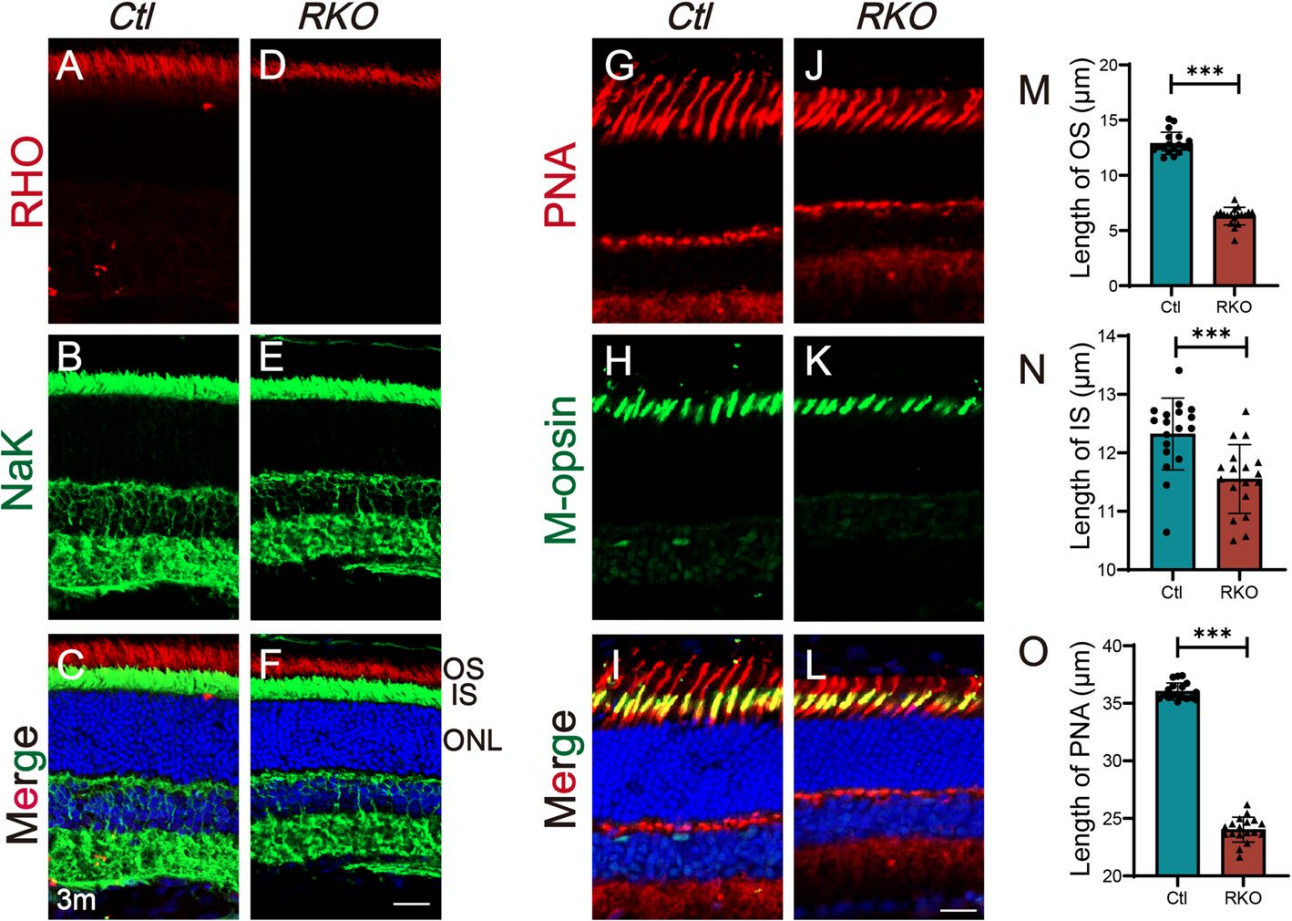
Immunofluorescence staining of retinal sections from 3-month-old mice. **A**–**F** Immunofluorescence staining of retinal rod cells from 3-month-old mice. RHO, red; NaK, green; DAPI, blue. Scale bar, 20 μm. OS, outer segment; IS, inner segment; ONL, outer nuclear layer. **G**–**L** Immunofluorescence staining of retinal cone cells from 3-month-old mice; M-opsin is shown in green, PNA is shown in red, and DAPI is shown in blue. Scale bar, 20 μm. **M**–**O** Statistical analysis of the lengths of the OS, IS, and PNA (indicated by the PRS of the cone cell). The lengths of the OS, IS, and PNA were measured using Zeiss software on the retinal sections at a distance of approximately 1000 μm from the optic centre (both dorsal and ventral sides). Number of mice, *n* = 3 for Ctl, and *n* = 3 for RKO. Three sections from each mouse were randomly collected. ***P < 0.001. Student’s *t-*tests were used for statistical analysis. All data are shown as mean ± SD

Rod cells make up approximately 97% of the photoreceptor cells in the mouse retina, and most IRD patients first present with rod degeneration. The outer segment (OS) of the rod is also called the disc membrane, which is critical for photon reception. Staining with a rhodopsin (RHO) antibody showed that the OS of retinas from the RKO mice decreased slightly at 2 months of age, and this phenotype became more severe at 3 months of age (Additional file 1: Fig. S5, Fig. 4A-F, M). The inner segment (IS, labelled with NaK), which is mainly responsible for the synthesis of numerous photoreceptor proteins, also became progressively shorter in the RKO mice (Additional file 1: Fig. S5, Fig. 4A-F, N). The cone is another photoreceptor cell type that is primarily responsible for colour recognition and visual accuracy in light situations. We used M-opsin and PNA (peanut agglutinin, which binds to cone matrix sheaths) to stain cone cells and showed that the cones of the RKO mice also progressively degenerated (Additional file 1: Fig. S5; Fig. 4G–L, O).

### Loss of PCYT1A compromises LD biogenesis without affecting overall PC synthesis

**Fig. 5:**
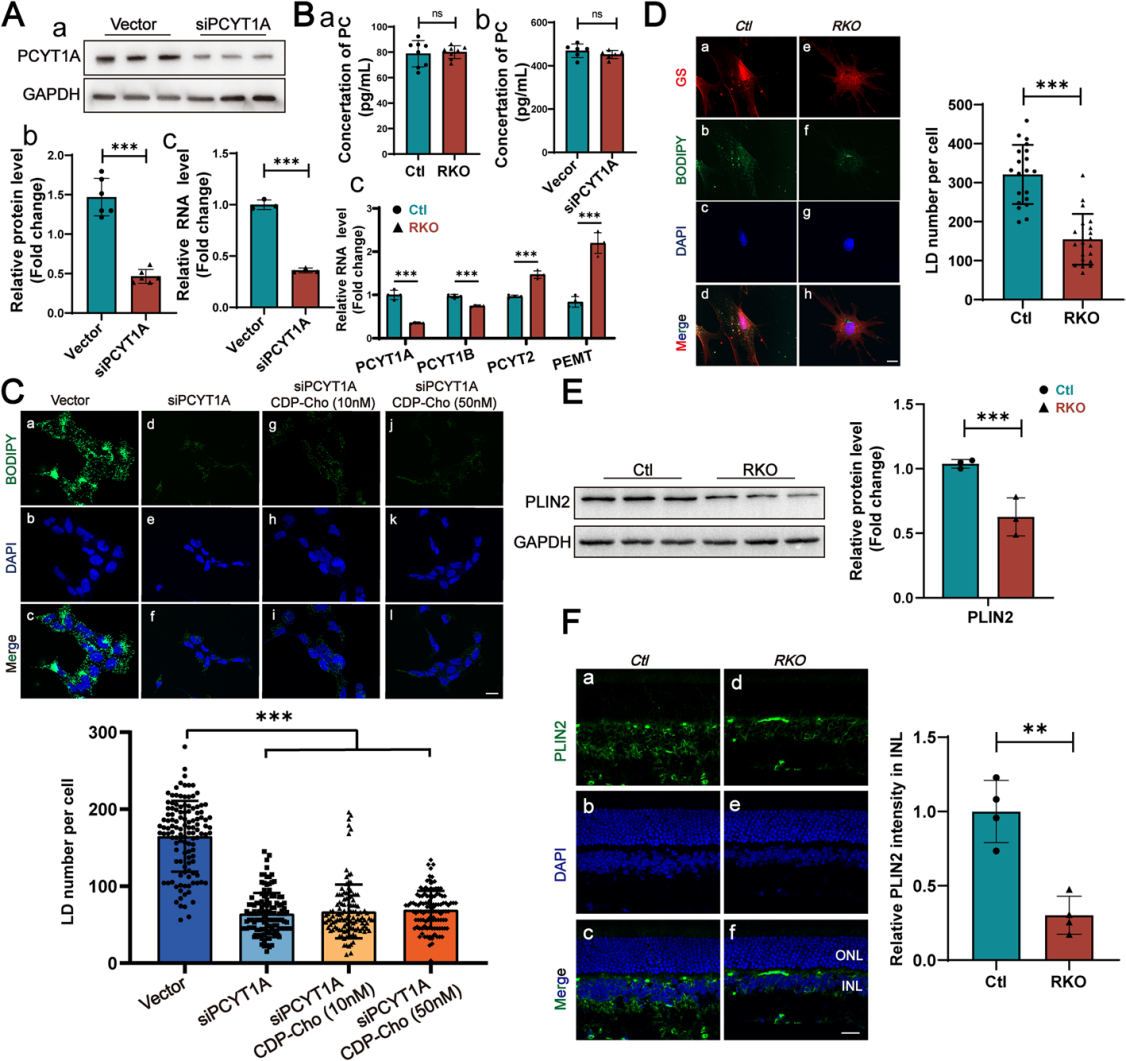
PCYT1A deficiency leads to impaired LD formation. **A** (a) and (b) Quantification of PCYT1A expression level in siPCYT1A ARPE-19 cells (*n* = 6) or vector cells (*n* = 6). (c) RT-qPCR shows relative RNA expression level of pcyt1a in siPCYT1A ARPE-19 cells (*n* = 3) or vector cells (*n* = 3). ***P < 0.001 by Student’s* t*-test. **B** (a) PC content analysis in retinas from 2-month-old RKO (*n* = 8) and Ctl (*n* = 8) mice. ns, not significant according to Student’s *t-*test. (b) PC content analysis in siPCYT1A ARPE-19 cells (*n* = 6) or vector cells (*n* = 6). ns, not significant according to Student’s *t-*test. (c) RT-qPCR assays of the RNA levels of key enzymes in PC synthesis pathways from 2-month-old RKO (*n* = 4) and Ctl (*n* = 4). ***P < 0.001 by multiple Student’s *t-*test. **C** Knockdown of PCYT1A in ARPE-19 cells reduced LD formation, and additional CDP-choline supplementation failed to rescue LD formation (120 cells from 3 independent experiments were counted for every group). BODIPY, green; DAPI, blue. Scale bars, 5 μm. ***P < 0.001 by multiple Student’s *t*-test. **D** Primary MG cells isolated from RKO mice showed compromised LD formation. GS (marker of MG cells) is shown in red, BODIPY is shown in green, and DAPI is shown in blue. Scale bar, 5 μm (20 cells for Ctl or RKO group were counted). ***P < 0.001 by Student’s *t*-test. **E** WB results showing reduced PLIN2 expression in retinas from 2-month-old Ctl (*n* = 3) and RKO (*n* = 3) mice. **P < 0.01 by Student’s *t*-test. **F** Immunofluorescence staining of retinas with an anti-PLIN2 antibody showed reduced PLIN2 protein levels from 2-month-old Ctl (*n* = 4) and RKO (*n* = 4) mice. Fluorescence intensity was measured using imageJ and threshold parameters were used as default. **P < 0.01 by Student’s *t-*test. All data are shown as mean ± SD

Given the well-established role of PCYT1A in PC synthesis, we hypothesized that PC deficiency may underlie the retinal degenerative phenotype observed in retinas from the RKO mice. Using lentivirus carrying shRNA targeting the *PCYT1A* gene, we knocked down the expression of PCYT1A in ARPE-19 cells. The PCYT1A protein level in the siPCYT1A cells was only approximately 30% of that in the control cells (Fig. 5A (a, b)), and PCYT1A mRNA assays showed the same results (Fig. 5A (c)), indicating that the shRNA was efficacious. Then, we separately examined the PC contents of the siPCYT1A and control cells and found no significant difference in PC levels between the two groups (Fig. 5B (a)). We also performed retinal PC content assays on 2-month-old RKO mice and their littermates and demonstrated that overall PC synthesis was not compromised under PCYT1A deletion conditions (Fig. 5B (b)). This finding could be due to compensatory activation of the PE methylation pathway: as shown in Fig. 5B (c), PCYT2 and PEMT mRNA were overexpressed in the retinas of the RKO mice. Another possible reason is that PCYT1B is involved in PC synthesis. The above experiments suggested that the retinal degeneration in the RKO mice was not simply caused by PC deficiency.

Recent studies have shown that PCYT1A regulates LD biosynthesis [10], although the exact molecular mechanism involved has not been fully elucidated. For this reason, we investigated whether the retinal degenerative phenotype was associated with the role of PCYT1A in the regulation of LD formation. By conducting LD formation experiments in the ARPE-19 cell line, we verified that the knockdown of PCYT1A indeed reduced LD biogenesis in the presence of OA (Fig. 5C). As PCYT1A was highly expressed in MG cells in the mouse retina, we also isolated primary retinal MG cells and carried out the same experiment. The results showed that LD formation was also compromised in MG cells from the RKO retinas (Fig. 5D).

Perilipin 2 (PLIN2) is a structural component of LDs that is required for their formation and maintenance. Therefore, we performed quantitative analyses of PLIN2 in the retinas of 2-month-old RKO and Ctl mice. The protein expression levels of PLIN2 were notably reduced (Fig. 5E). Immunofluorescence staining for PLIN2 is often used as an indicator of LDs. We stained retinal sections with an anti-PLIN2 antibody and found that PLIN2 was predominantly expressed in the outer plexiform layer (OPL), INL, and inner plexiform layer (IPL) but not in the classic LDs (Fig. 5F). Quantitative fluorescence analysis of the INL revealed reduced PLIN2 expression in the INL (Fig. 5F), which was consistent with the WB results shown in Fig. 5E. Unlike those of zebrafish or nematodes, higher mammalian retinal tissues do not normally store lipids in LDs, so the extent to which the impaired function of PCYT1A in LD formation is associated with the retinal degenerative phenotype is still an open question and will be discussed later.

### Proteomic analysis reveals aberrant protein expression in fatty acid metabolism and ferroptosis signalling pathways

**Fig. 6:**
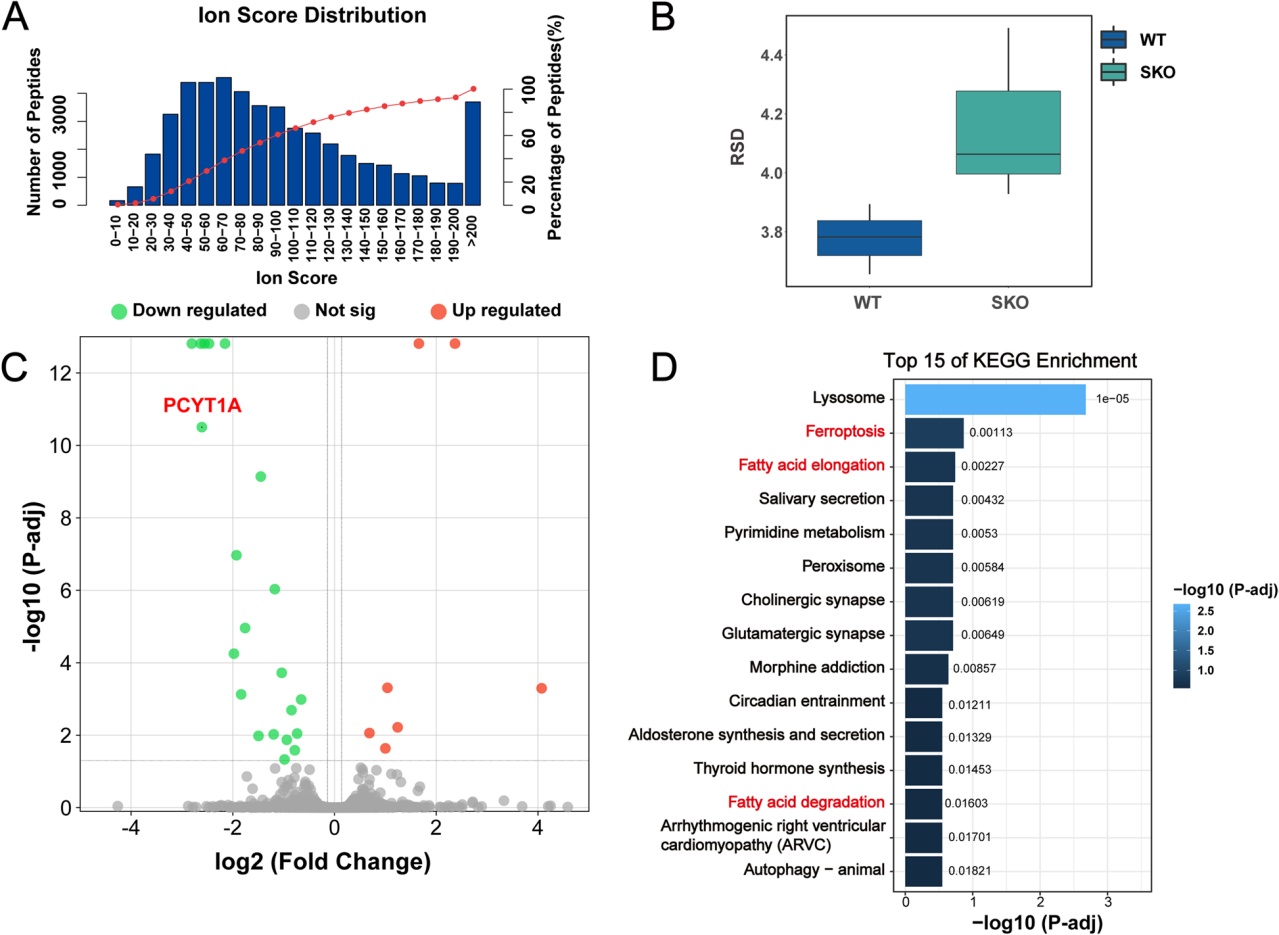
Proteomic analysis of retinal protein expression in RKO and Ctl mice. **A** Ion score distribution indicating that approximately 70% of the peptides scored above 60. **B** Relative standard deviations of retinal proteins in 2-month-old mice. **C** Volcano plot showing the DEPs in the mouse retina. Downregulated proteins are represented by green dots, upregulated proteins are represented by red dots, and proteins with no significant differential expression are represented by grey dots. The thresholds were set at *P* (adjusted) < 0.05 and log2 (fold change) > 1.2. **D** KEGG pathway analysis of the DEPs enriched in different pathways, showing the top 15 ranked pathways

To further elucidate the mechanisms by which PCYT1A deficiency leads to photoreceptor degeneration, we employed a 4D label-free approach to analyse the global proteomic landscape of retinas from 2-month-old RKO and littermate Ctl mice [33]. As shown in the volcano plot, a total of 28 differentially expressed proteins (DEPs) were observed, among which 21 proteins were downregulated and 7 proteins were upregulated (Fig. 6C, Additional file 2: Table S1). Our peptide ion scores and relative standard deviation results showed that the proteomic results had little sample variation and stable results (Fig. 6A, B). Furthermore, a marked reduction in the abundance of PCYT1A was observed (Fig. 6C). KEGG pathway analysis revealed that the DEPs were mainly clustered in the lysosome pathway, ferroptosis pathway, fatty acid elongation pathway, salivary secretion pathway, and fatty acid degradation pathway (Fig. 6D). Lysosomal pathways are associated with the synthesis and degradation of almost all proteins and are not considered specific; therefore, the DEPs were clustered into ferroptosis pathways, fatty acid elongation pathways, and fatty acid degeneration pathways.

### PCYT1A deficiency triggers lipid peroxidation and glutathione depletion, leading to ferroptosis

**Fig. 7:**
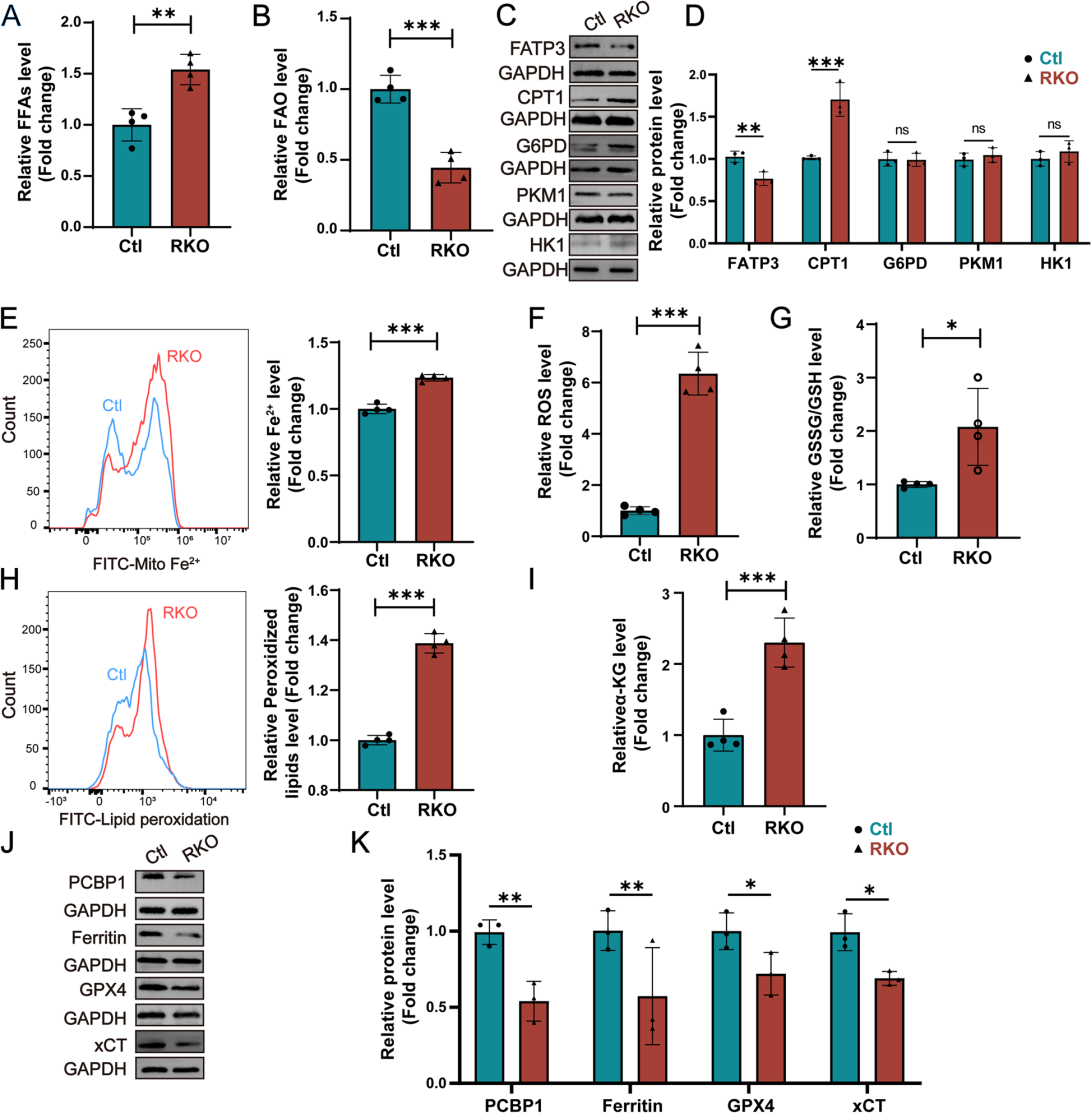
Deletion of PCYT1A causes FFA accumulation and ferroptosis in the mouse retina. **A** FFA accumulation in the retina of RKO (*n* = 4) mice compared to the Ctl (*n* = 4) group. **P < 0.01 by Student’s *t*-test. **B** Reduced FAO levels in the retinas of RKO mice (*n* = 4) compared to the Ctl (*n* = 4) group. ***P < 0.001 by Student’s *t*-test. **C** Expression of proteins involved in fatty acid metabolism in the retinas of 2-month-old Ctl (*n* = 3) and RKO (*n* = 3) mice. **D** This panel shows the quantification of the proteins expression levels. The expression of each protein was normalized to that of GAPDH. **P < 0.01, ***P < 0.001, ns, not significant according to multiple Student’s *t*-tests. **E** The mitochondrial Fe^2+^ concentration was significantly increased in the retinas of 2-month-old RKO (*n* = 4) mice compared to the Ctl (*n* = 4) group. Flow cytometry peak plots are shown on the left; the blue curve represents the Ctl group, while the red curve represents the RKO group. Relative quantitative statistics are plotted on the right. ***P < 0.001 by Student’s *t*-test. **F**–**G** ROS and GSSG/GSH levels were increased in the retinas of 2-month-old RKO (*n* = 4) mice compared to the Ctl (*n* = 4) group. *P < 0.05, ***P < 0.001 by Student’s *t*-test. **H** Retinal lipid peroxidation levels were increased in the retinas of 2-month-old RKO (*n* = 4) mice compared to the Ctl (*n* = 4) group. Flow cytometry peaks are shown on the left; the blue curve represents the Ctl group, and the red curve represents the RKO group. Relative quantitative statistics are plotted on the right. ***P < 0.001 by Student’s *t*-test. **I** Retinal α-KG levels were increased in retinas of 2-month-old RKO (*n* = 4) mice compared to the Ctl (*n* = 4) group. ***P < 0.001 by Student’s *t*-test. **J** Expression analysis of proteins involved in the ferroptosis pathway in retinas of 2-month-old Ctl (*n* = 3) and RKO (*n* = 3) mice. **K** Quantification of the protein expression levels. The expression of each protein was normalized to that of GAPDH. *P < 0.05, **P < 0.01 by multiple Student’s *t*-tests. All data are shown as mean ± SD

As the proteomic results suggested an abnormal fatty acid metabolic pathway, we further examined the levels of FFAs and FAO in the retinas of the RKO mice. We showed that retinal FFAs were elevated in the RKO mice compared to the Ctl mice at 2 months of age (Fig. 7A). Reduced FFA consumption appeared to be the cause of excessive FFA accumulation, as indicated by reduced FAO in the retinas of the RKO mice (Fig. 7B). These results suggested that the FFA utilization of the cellular mitochondria was impaired. After the uptake of FFAs, cells activate the carnitine cycle and β-oxidation (FAO), which ultimately provides energy through the tricarboxylic acid cycle (TCA cycle) and oxidative phosphorylation [34]. Therefore, we further examined the expression of the key enzymes fatty acid transporter 3 (FATP3, a member of the FATP family) and carnitine palmitoyltransferase 1 (CPT1, a rate-limiting enzyme) during the fatty acid uptake and FAO processes. WB results showed that the expression of the FATP3 protein was reduced, while that of CPT1 was elevated (Fig. 7C, D). These data indicate a deficiency in the ability of fatty acids to function effectively within cellular mitochondria. However, further investigation is required to determine how PCYT1A regulates this process. A reasonable explanation is that excessive FFA accumulation in the cytoplasm inhibits the cellular uptake of FFAs and facilitates their translocation from the cytoplasm to the mitochondria. We also examined the expression levels of glucose-6-phosphate dehydrogenase (G6PD), pyruvate kinase M1 (PKM1), and hexokinase-1 (HK1), which are key enzymes in glucose metabolism, and found that these proteins were not significantly affected (Fig. 7C, D), demonstrating that PCYT1A deficiency mainly affects lipid metabolism.

Proteomic data suggested that the ferroptosis pathway in the retinas from the RKO mice is aberrantly activated; thus, we examined the main indicators related to ferroptosis. We found that the levels of mitochondrial Fe^2+^, ROS, GSSG/GSH, and peroxidized lipids were elevated in the retinas of the RKO mice (Fig. 7E–H), suggesting that ferroptosis was activated in the retinas of these mice. α-KG in the mitochondrial TCA cycle can also stimulate dihydrolipoamide dehydrogenase (DLD) to produce mitochondrial ROS and increase cellular iron levels involved in the ferroptosis pathway, and studies have shown that elevated α-KG can cause ferroptosis [35, 36]; therefore, we examined α-KG levels in the retina. The results showed that α-KG levels were significantly elevated in the retinas of the RKO mice, further supporting the occurrence of ferroptosis (F ig. 7I). Excessive accumulation of ROS can also cause imbalances in cellular energy metabolism and is one of the reasons for ferroptosis. The cystine/glutamate reverse transporter (System Xc-) is an important antioxidant system for cells that takes up cystine and excretes glutamate. The cystine that enters the cell is reduced to cysteine, which is involved in the synthesis of GSH. GSH reduces toxic lipid peroxides to nontoxic fatty alcohols under the catalytic action of GPX4. This process uses GSH as the reducing agent and GPX4 as the key enzyme that mediates the reduction reaction of lipid peroxides and negatively regulates ferroptosis. GSH depletion leads to the accumulation of toxic peroxides, damage to proteins and cell membranes, and subsequent ferroptosis. We examined the levels of xCT (cystine transporter protein) and GPX4 (ferroptosis inhibitor protein) in the retinas of mice by WB. We found that in the retinas of the RKO mice, the xCT and GPX4 protein levels were significantly lower and the oxidized glutathione level was greater than those in the retinas of the Ctl mice (Fig. 7J, K). In the ferroptosis process, iron (Fe^2+^) is required for the accumulation of lipid peroxides, a key event in the occurrence of iron death. Therefore, iron uptake, transport, and storage can regulate ferroptosis. The maintenance of iron homeostasis in living organisms is tightly regulated. The cytoplasmic molecular chaperone of iron PCBP1 is a multifunctional RNA-binding protein involved in gene transcription, RNA regulation, and iron loading into ferritin (FTH1). FTH1 is an iron storage protein, and intracellular Fe^2+^ can be processed into FTH1 for storage by PCBP1. To this end, the protein levels of PCBP1 and FTH1 were examined, and we found that both proteins were significantly downregulated after PCYT1A deletion in the retinas of the RKO mice at 2 months of age (Fig. 7J, K), which indicated that the decrease in cellular iron storage proteins increased free iron and induced ferroptosis via cellular iron overload. The increased levels of mitochondrial Fe^2+^ in the retinas of the RKO mice also confirmed the presence of Fe^2+^ overload (Fig. 7E). By using transmission electron microscopy, we observed the typical ferroptosis phenomena of mitochondrial ridge disappearance and breakage in the INL layers of the retina from the RKO mice (Additional file 1: Fig. S6).

**Fig. 8:**
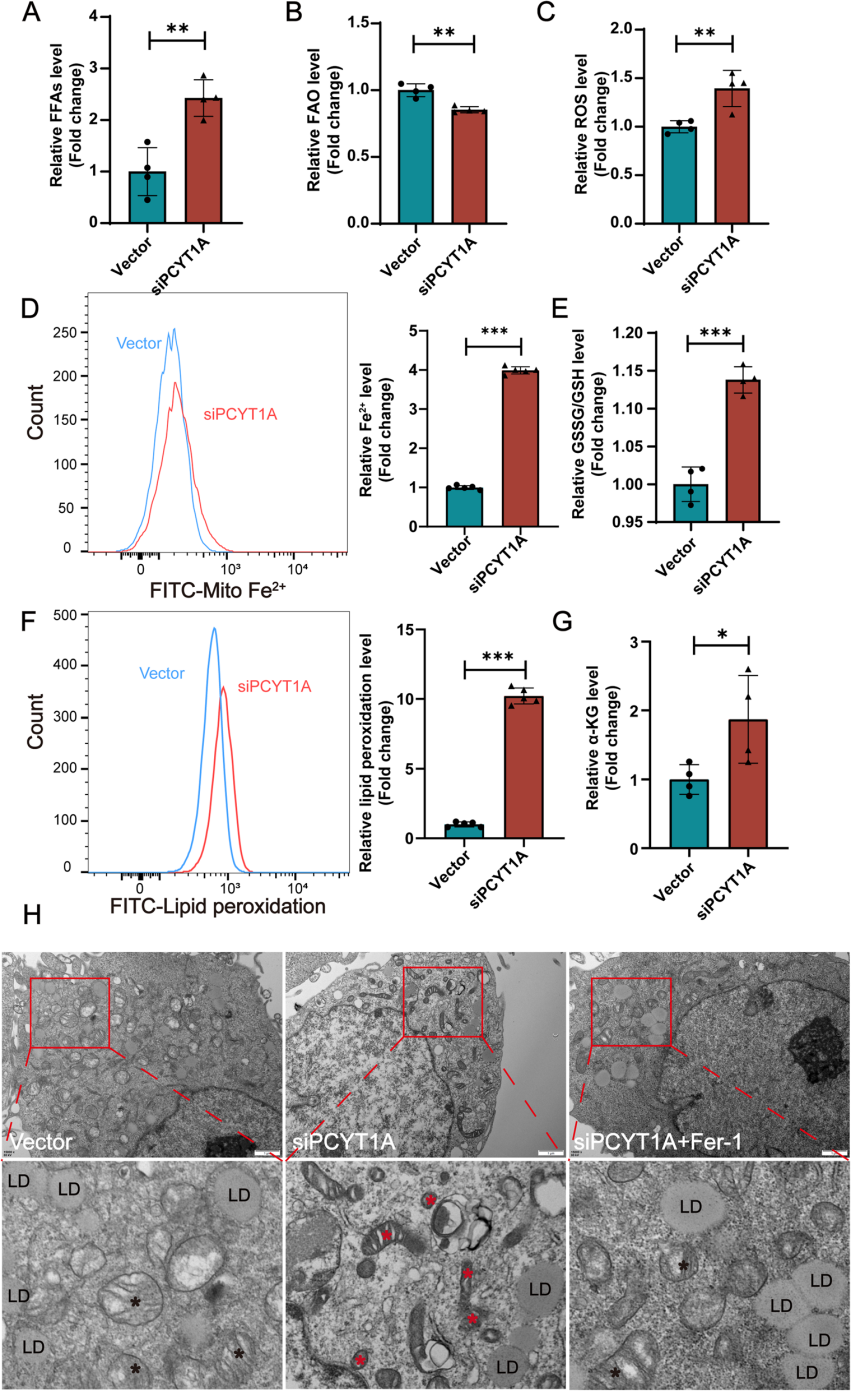
Knockdown of PCYT1A in ARPE-19 cells leads to ferroptosis in the presence of OA. **A** FFA levels were increased in the siPCYT1A (*n* = 4) group compared to vector (*n* = 4) group. Student’s* t*-test. **B** Reduced FAO levels in the siPCYT1A group (*n* = 4) compared to vector (*n* = 4) group. Student’s *t-*test (**C**) ROS was increased in the siPCYT1A (*n* = 4) group compared to vector (*n* = 4) group by Student’s *t-*test. **D** Mitochondrial Fe^2+^ was increased in the siPCYT1A (*n* = 5) group compared to vector (*n* = 5) group. **E** GSSG/GSH was increased in the siPCYT1A (*n* = 4) group compared to vector (*n* = 4) group. **F** Peroxidized lipids were increased in the siPCYT1A (*n* = 5) group compared to vector (*n* = 5) group. **G** α-KG was increased in the siPCYT1A (*n* = 4) group compared to vector (*n* = 4) group. **H** Representative transmission electron microscopy images of ARPE-19 cells revealing that the mitochondria in the siPCYT1A group exhibited substantial wrinkling, reduced mitochondrial ridges, and increased membrane density. In contrast, the mitochondria of the siPCYT1A cells were rescued after Fer-1 treatment (a 1 mM master batch was prepared with DMSO at a working concentration of 40 μM). Student’s *t*-tests were used for statistical analysis. *P < 0.05, **P < 0.01, ***P < 0.001. Asterisks indicate the mitochondria. Scale bar, 500 nm. All data are shown as mean ± SD

The above results demonstrated that dysregulated FFA metabolism and ferroptosis occurred in the RKO retina, but which process occurred earlier? To answer this question, we used ARPE-19 cells to investigate whether the ferroptosis pathway was activated in both OA-treated (OA + , Fig. 8) and OA-free (OA − , Additional file 1: Fig. S7) conditions. In OA + conditions, FFAs accumulated in the siPCYT1A cells (Fig. 8A), and the FAO level was also reduced (Fig. 8B). Ferroptosis indicators, including ROS, mitochondrial Fe^2+^, GSSG/GSH, peroxidized lipids and α-KG, were increased in the PCYT1A knockdown cells (Fig. 8 C–G). These results were similar to the findings in the retinas of the RKO mice (Fig. 7). We also observed that the mitochondria in the siPCYT1A group exhibited substantial wrinkling, reduced mitochondrial ridges, and increased membrane density (Fig. 8H). Ferrostatin-1 (Fer-1) is a classic ferroptosis inhibitor that inhibits lipid peroxidation [37]. We found that the changes in mitochondrial morphology caused by PCYT1A knockdown were largely reversed by Fer-1 (Fig. 8H), further confirming that ferroptosis indeed occurred in the siPCYT1A cells. However, under OA − conditions, we only detected elevated levels of ROS and FAO, while the levels of key indicators of ferroptosis, such as mitochondrial Fe^2+^, GSSG/GSH, and peroxidized lipids, did not change (Additional file 1: Fig. S7). These results suggested that in the absence of excess FFAs, siPCYT1A cells exhibited accelerated mitochondrial oxidation, which is not sufficient to cause ferroptosis; therefore, the occurrence of ferroptosis is dependent on the disruption of excess FFA metabolism.

## Discussion

IRDs are among the most complicated inherited diseases and show substantial genetic and clinical heterogeneity. Therefore, the pathogenic mechanisms involved are extraordinarily complex, and abnormalities in the phototransduction cascade [21, 38, 39], vitamin A metabolism [40, 41], the cytoskeleton [42], cell adhesion [43], RNA splicing [44], and protein trafficking are the main known causes of the development of IRDs [45]. Generating appropriate animal models and determining the underlying pathogenic mechanisms are essential to understanding the pathogenesis of this disease.

Mutations in PCYT1A, the key enzyme in PC biosynthesis via the Kennedy pathway, are associated with IRDs, but the pathological mechanism is still unclear. Given the critical function of PCYT1A in PC synthesis, it is easy to attribute the occurrence of IRDs to PC deficiency. However, we are puzzled by the fact that there is another alternative pathway for PC synthesis and that PC synthesis in the retina is low. In this study, we found that PCYT1A was mainly expressed in the INL in the mouse retina (Fig. 1 C–G), in contrast to reports that PCYT1A is mainly expressed in the retinal ONL [2]. Upon careful review of the immunofluorescence staining results from Afreen Haider et al., we found that PCYT1A showed obvious staining in both the INL and RGC layers, which is similar to our results. In addition, the degenerative phenotype in the INL of the RKO mice validated PCYT1A expression in the INL. Since *PCYT1A* mutations also cause isolated IRDs [15], we investigated the function of PCYT1A in the retina. To this end, we established a mouse model with *Pcyt1a* knockout in the retina (RKO) by using the Cre-loxP system (Additional file 1: Fig. S2). Phenotypic studies revealed that the RKO mice displayed retinal degenerative features, such as reduced ERG response, decreased retinal thickness, and photoreceptor loss [13–15], similar to what has been observed in IRD patients with *PCYT1A* mutations (Figs. 2, 3, and 4). These findings support previous genetic studies and suggest that PCYT1A plays essential roles in the retina. In addition, retinal photoreceptor dystrophy in the RKO mice appears to occur later than the loss of MG cells in the INL layer (Figs. 2 and 3). This result is consistent with our finding that PCYT1A is mainly expressed in the MG cells of the retina. The MG cells are the predominant glial element in the retina, representing 90% of retinal glia [46]. Most nutrients, waste products, ions, water, and other molecules are transported through the MG cells between vessels and neurons [47] and play critical roles in glia–neuron interactions and retinal homeostasis. We observed photoreceptor cell degeneration secondary to INL loss in the RKO mice, which may be due to the disruption of the interaction between photoreceptors and MG cells. However, how the deletion of PCYT1A disrupts this interaction needs to be further investigated.

Unsurprisingly, we did not observe a significant decrease in overall PC in the retinas from the RKO mice (Fig. 5), as the PE methylation pathway was activated in compensation, as indicated by the increased expression of PCYT2 and PEMT (Fig. 5). Another reason could be the uptake of PC or its synthetic precursors from the circulatory system by the retina. The unchanged PC content made it difficult to interpret subsequent mechanistic studies. We found that PCYT1A knockdown compromised LD formation in ARPE-19 cells and isolated primary MG cells after OA supplementation. In the retina, we also observed decreased expression of PLIN2 proteins, which are indicators of LDs. Unfortunately, we did not directly observe LDs in the retina, either in the RKO mice or the Ctl mice. This finding may be because under normal conditions, retinal cells do not store lipids, and LDs may only appear under stressful or genetic defect conditions in mice, in contrast to the results in scleractinian fishes or other lower organisms [48–51]. In addition, we observed substantial expression of PLIN2 in the INL layer of the retina but not in the form of LDs. Whether this phenomenon is involved in the regulation of lipid metabolism in ways other than forming LDs is not known and deserves further investigation.

In the present study, we also found that CDP-choline supplementation could not rescue LDs that were reduced due to PCYT1A knockdown (Fig. 5). We suggest two possible reasons: (1) The involvement of PCYT1A in LD formation does not depend on its enzymatic activity but rather on the process of interaction between the protein itself and LDs. (2) LD formation depends on the ability of PCYT1A to synthesize PC de novo in a short time period without affecting the overall PC content of the cells. Recent studies in the brain have shown that under hyperactivation conditions, neurons produce large amounts of toxic fatty acids, which astrocytes can use and consume, thus protecting neurons from damage [52]. Since the retina and the brain are part of the central nervous system (CNS) and MG cells and astrocytes share many similarities [53], whether this coupling system or other similar operation mechanisms are also present in the retina needs to be further clarified.

Ferroptosis, a type of cell death caused by iron-dependent lipid peroxidation, was recognized as a distinct phenomenon and given its name a decade ago. Ferroptosis is associated with a wide range of biological contexts, including development, ageing, immunity, and cancer [54]. Ferroptosis differs from apoptosis and other forms of cell death [55]. Several studies have shown the relationship between iron metabolism and IRDs. In mice with targeted mutations in the iron-exporter ceruloplasmin, retinal iron overload occurs at an age-dependent rate, leading to degeneration of the retina that shares features with age-related macular degeneration (AMD) [56]. Research revealed that in rd10 mice, elevated levels of transferrin and transferrin receptors were accompanied by elevated levels of ferritin and ferritin-bound iron, indicating that retinal degeneration may be linked to altered iron homeostasis [57]. In addition, several relevant studies have shown the link between lipid metabolism and retinal diseases. Xin Li et al*.* reported that fatty acid elongase 2 (ELOVL2) increases endoplasmic reticulum stress by regulating lipid metabolism and that mitochondrial dysfunction leads to an AMD phenotype in RPE cells [58]. Another study reported that excess circulating lipids inhibited retinal autophagy in a *Vldlr − / − *angiomatous proliferation model, which contributed to retinal pathological angiogenesis associated with AMD disease pathogenesis [59]. However, there is no conclusive evidence that fatty acid metabolism and ferroptosis are associated with IRDs, and our study demonstrated the link between these processes, providing a new understanding of the pathogenesis of IRDs.

## Conclusions

In summary, our findings revealed that ferroptosis induced by PCYT1A knockdown was dependent on excess FFAs, and in the RKO retina, ferroptosis occurred mainly in the INL layer, suggesting that MG cells may be involved in the catabolism of FFAs in the retina. Furthermore, the recovery of cells to a healthy mitochondrial state via Fer-1 treatment further indicates that PCYT1A deficiency results in cellular ferroptosis, and this molecule may be a potential therapeutic target for IRDs.

## Methods

### Mouse model and cell lines

All animal-related experiments were approved by the Animal Care and Use Committee of Sichuan Provincial People’s Hospital and were performed in accordance with the Declaration of Helsinki. All experimental procedures were carried out strictly in accordance with the approved study protocols. The mice were raised under regular light conditions with a 12-h light/12-h dark cycle and unrestricted access to food and water. Euthanasia was performed by dislocating the cervical vertebrae of mice after anaesthesia with 1% sodium pentobarbital anaesthetic (at a dose of 10 μL/g body weight).

*Pcyt1a*^*loxP/*+^ (*Pcyt1a*^*fl/*+^) transgenic mice on a C57BL/6 J background were generated by Cyagen Biosciences (Suzhou, China). By employing the CRISPR/Cas9 approach, we generated conditional *Pcyt1a* knockout mice by replacing exons 4–5 of *Pcyt1a* with a homologous region containing two loxP sites. This modification resulted in a frameshift in the *PCYT1A* transcript and caused the loss of function of the *Pcyt1a* gene. The primer pairs F1 (5′-CTAACTACAAGACGCTTTCCCCA-3′) and R1 (5′-GCTTCAGTCCCTTGGCTTTAGAA-3′) were used to identify the upstream loxP sequence.

To prevent off-target effects caused by CRISPR/Cas9, we crossed *Pcyt1a*^*fl/*+^ mice with C57BL/6 J mice for six generations before mating them with Six3-Cre mice. Genotyping of the *Six-Cre* transgenic mice was carried out by PCR using Six-F (5′-GAACGCACTGATTTCGACCA-3′) and Six-R (5′-GCTAACCAGCGTTTTCGTTC-3′). The HEK293T and ARPE-19 cell lines were purchased from ATCC and cultured in DMEM (Gibco, USA) at 37 °C in a 5% CO_2_ incubator. For knockdown of the *PCYT1A* gene, ARPE-19 cells were transduced with a lentivirus carrying shRNA targeting *PCYT1A* (5′-CCCTTTCTGTCCCATTACCTT-3′, Genechem, Shanghai, China) or negative control shRNA (5′-TTCTCCGAACGTGTCACGT-3′) according to the manufacturer’s protocol. The use of lentiviruses in this study was approved by the Institutional Biosafety Committee of Sichuan Provincial People’s Hospital.

### RNA isolation, reverse transcription–polymerase chain reaction (RT-PCR), and RT-qPCR

Mouse retinal tissue RNA was isolated using TransZol Up reagent, and reverse transcription was subsequently performed using EasyScript All-in-One First-Strand cDNA Synthesis SuperMix for qPCR. The primer pairs used for different genes are listed in Additional file 2: Table S2. Target gene expression was normalized to GAPDH mRNA expression.

### Single-cell RNA-seq

#### Sample preparation

Experiments were conducted using 3-month-old wild-type C57BL/6 J mice. For isolation of viable single cells from retina/retinal pigment epithelium (RPE)/choroid tissue, the mice were euthanized, and the eyes were enucleated for further processing. Each eye was dissected under a stereomicroscope to remove the anterior segment, including the cornea, lens, iris, and ciliary body. The neural retina and RPE/choroid tissue were gently scraped from the sclera. After PBS rinses, the tissue was transferred to tissue storage solution and sent to Genergy Bioscience Co., Ltd. (Shanghai), for further processing.

#### Normalization of low-mass cell filtration and gene expression data

Low-quality cells were filtered using Seurat software. By default, cells with gene counts greater than or equal to 200, mitochondrial genes with unique molecular identifier (UMI) sequence counts less than or equal to 10%, erythrocyte marker genes with UMI sequence ratios less than or equal to 10%, or bicellular cells identified by the Scrublet software were considered low-quality cells and were filtered and removed. For the remaining high-quality cells, we used Seurat’s NormalizeData function to normalize the gene expression counts of each cell under the default parameters.

#### Cluster analysis of samples

For the number of UMI sequences of high-quality single cells and genes within the sample, the total number of UMIs per cell and the scaling factor ratio of 10,000 were calculated to correct for the depth of cellular sequencing to perform a standardized normalization process. Based on the diffusion coefficient measure of the high degree of variability of genes, the similarity between the cells was determined using principal component analysis (PCA) downscaling, and the closer the distance of the samples was, the closer the expression trend of the genes was. The top 30 principal components with the largest variance in the PCA results were visualized by the reduction method t-distributed stochastic neighbour embedding (TSNE). Seurat was used to separately cluster high-quality populations of cells. The PCA space was used to construct a nearest neighbour KNN map based on Euclidean distances, and then, the Louvain modularity optimization algorithm was used to cluster the cell populations.

#### Cell type discrimination analysis

The FindAllMarkers function was used to identify specific marker genes for a cell subpopulation. This process was performed by sequentially comparing cells of a specific cell subpopulation with all other cells by the Wilcoxon rank sum test to identify differentially expressed genes in that cell subpopulation. A Bonferroni-corrected *P* value less than 0.05 was used as a threshold to define statistically significant differentially expressed genes. Genes whose average expression in a specific cell subpopulation was more than twofold greater than the average expression in other cell subpopulations were selected as marker genes. The cell type to which each cell subpopulation belonged was annotated using previously reported passive cell type-related marker genes and the top-ranked differentially expressed genes. Detailed information on the retinal cell clusters is provided in Additional file 2: Table S3.

### ERG recordings

As outlined in our previous study, ERG recordings were obtained [17, 60]. Over the course of the night, the mice underwent dark adaptation, and subsequent procedures were conducted in a dim-red light environment. Mice were subjected to deep anaesthesia via inhalation of a mixture of xylazine (80 mg/kg) and ketamine (16 mg/kg). To prepare the mice for the ERG test, we administered tropicamide, phenylephrine, and tetracaine (0.5%) to the mouse eyes before the procedure. Subsequently, the ERG responses of the mice were recorded and analysed using the Espion Visual Electrophysiology System from Diagnosis, LCC (Littleton, MA, USA). Dark-adapted ERGs were measured in response to varying intensities of light flashes.

### Optical coherence tomography (OCT)

Mice were anaesthetized with 1% sodium pentobarbital anaesthetic (at a dose of 10μL/g body weight) prepared in 0.9% saline, and tobramycin eye drops (Runzheng, China) were added to the cornea. After the pupils of the mice were allowed to dilate, an OCT scan was started, and images were collected according to standard OCT test procedures. The images were also assessed and processed using the accompanying image processing software.

### Immunohistochemical analysis

For retinal staining, the eyes of Pcyt1a-RKO (RKO) mice and control (Ctl) mice were collected, fixed in 4% paraformaldehyde for 2 h, and then soaked in 30% sucrose in PBS for another 2 h at 4 °C. Following lens removal, the eyes were inserted into an optimal cutting temperature (OCT) solution and sliced at a thickness of 12 μm. The sections were blocked and permeabilized with blocking buffer (5% FBS, 0.5% Triton X-100, 0.01% NaN_3_ in PBS) and then incubated with the primary antibody overnight at 4 °C. After being rinsed in PBS three times, the sections were incubated with Alexa Fluor 488/594-conjugated goat anti-rabbit/mouse secondary antibodies, and the nuclei were co-labelled with DAPI.

### Western blotting (WB)

For WB, samples were homogenized in standard RIPA lysis buffer containing Complete Protease Inhibitor Cocktail (Roche, Switzerland). For the determination of the protein concentration, an enhanced BCA protein assay kit was used. Standard SDS–PAGE procedures were followed. The samples were blocked with 8% skim milk in TBST buffer for 2 h at room temperature before being incubated with primary antibodies in blocking solution overnight at 4 °C. Signals were developed using SuperSignal West Pico Chemiluminescent Substrate (Thermo Fisher, USA). The relevant antibody information is listed in Additional file 2: Table S4. Uncropped blots are shown in Additional file 3. ImageJ was used to determine the relative protein density.

### LD formation analysis

Retinal primary Müller cells and ARPE-19 cells were collected after induction with 50 μM oleic acid (OA) (fatty acid-free bovine serum albumin: OA dissolved in EBSS at a ratio of 6:1) for 3 h. LDs were labelled with BODIPY 493/503 (catalogue number: D3922, Sigma, USA). Each labelled LD was automatically counted using Image-Pro Plus software. The statistical analysis was then carried out using independent samples or multiple Student’s *t-*test with GraphPad Prism 8.0 software.

### Primary Müller glial (MG) cell isolation

A 24-well plate was lined with round coverslips and washed three times with PBS for 5 min. Then, 500 μL of poly-L-lysine (catalogue number: P4707, Sigma, USA) was added, and the plate was incubated overnight in a cell incubator. Poly-L-lysine was recovered, and 200 μL of laminin (catalogue number: L2020, Sigma, USA) was added. PBS diluted to a working concentration of 2 μg/cm^2^ was added to the well plates. Retinas from 12-day-old mice were rinsed with HBSS and then placed in 1 mL of preprepared papain (catalogue number: LS003119, Worthington, USA; working concentration: 20 U/mL). The tissue was processed into small pieces by slow aspiration 5 times with a 2.5-mL syringe. After treatment in the previous step, the tissue suspension was digested in an incubator at 37 °C for 30 min. One millilitre of DMEM (containing 10% FBS and 1% PS) was added to terminate the digestion, and the cells were filtered through a 40-μm nylon membrane. The filtered cell suspension was collected into a 15-mL centrifuge tube at 800 × *g* for 4 min. The cells were resuspended in MACS buffer (with serum in PBS) and washed again by centrifugation at 800 × *g* for 4 min. The supernatant was removed, and 1 mL of astrocyte medium was added for resuspension. The single-cell suspensions were added to a prewrapped 24-well plate and incubated at 37 °C in a 5% CO_2_ cell incubator. After 3 days, the cells were observed under a microscope for well attachment, and the medium was replaced with fresh astrocyte medium. After 7 days of culture, the cells were induced with OA (catalogue number: O1008, Sigma, USA) for 3 h. Samples were collected, and slices were prepared.

### Free fatty acid (FFA) detection

For quantification of FFAs in retinas or cells, an FFA detection kit (catalogue number: ml092765, Mlbio, Shanghai, China) was used. Retinas from 2-month-old mice were homogenized and centrifuged at 5000 × *g* and 4 °C for 5 min, and the organic phase was collected for measurement. For cells, an extraction solution was added, and the mixture was sonicated, shaken for 15 min, and centrifuged at 5000 × *g* for 5 min at 4 °C. The organic phase was collected for analysis. The sample was mixed with working solution and centrifuged at 4 °C and 5000 × *g* for 5 min. The upper organic phase was separated, reagent IV was added, and the absorbance at 550 nm was measured after 10 min. The content of FFAs was calculated using the standard curve.

### Reactive oxygen species (ROS) assay, fatty acid oxidation (FAO) assay, PC analysis, and α-ketoglutarate (α-KG) analysis

Two-month-old RKO mice and their littermate controls were chosen for this study. Retinas were isolated and homogenized, followed by centrifugation at 3000 × *g* for 10 min at 4 °C to obtain the supernatant for analysis. After treatment with 0.25% trypsin, the cells were harvested by centrifugation and lysed via cell sonication. The supernatant was collected for measurement after centrifugation at 3000 × *g* and 4 °C for 10 min. The experimental procedures were performed according to the manufacturer’s instructions for the kit used. Relevant information is included in Additional file 2: Table S4.

### Measurement of mitochondrial Fe^2+^

Retinal tissue was processed into a single-cell suspension using the same method used for primary MG cell culture. The level of mitochondrial Fe^2+^ was determined using Mito-FerroGreen (catalogue number: M489, Dojindo, Japan). After the culture medium was removed, the cells were washed three times with serum-free medium, stained with Mito-FerroGreen working solution at 37 °C and 5% CO_2_ for 30 min in a cell culture incubator, collected by centrifugation after removal of the supernatant, washed with PBS and resuspended. The fluorescence intensity was measured using a flow cytometer with an excitation wavelength of 488 nm and an emission wavelength of 520 nm.

### Analysis of lipid peroxides in cells

The cells were seeded into a 6-well plate and incubated overnight at 37 °C in a 5% CO_2_ incubator. After removal of the culture medium, the cells were washed twice with PBS and then treated with OA (50 μM) for 3 h before collection. For retinal tissue, the retinas of 2-month-old mice were dissected and prepared as single-cell suspensions. Working solution [prepared according to the instructions (catalogue number: L267, Dojindo, Japan)] was added to the prepared samples and incubated at 37 °C in a 5% CO_2_ incubator for 30 min. After the working solution was removed, the cells were washed twice with PBS. The cells were then digested, centrifuged, washed with PBS, and resuspended in 1 mL of PBS, after which the FITC fluorescence intensity was assessed using a flow cytometer (NovoSamplerQ, Agilent, USA).

### GSSG/glutathione (GSH) analysis

A GSSG/GSH kit (catalogue number: G263, Dojindo, Japan) was used to determine the GSSG/GSH ratio. Retinas from 2-month-old mice were homogenized in 600 μL of 5% SSA solution and centrifuged at 8000 × *g* for 10 min, after which the supernatants were collected. The SSA concentration was adjusted to 0.5% with ddH_2_O. The cells were collected at 200 × *g* for 10 min at 4 °C, lysed with 80 μL of 10 mM HCl, and subjected to two cycles of freezing/thawing. Then, 20 μL of 5% SSA was added, and the mixture was centrifuged at 8000 × *g* for 10 min. The reaction mixture was prepared according to the kit instructions, and the absorbance was measured at 405 nm.

### Proteomic analysis

#### Sample processing and collection

Three 2-month-old RKO mice and three littermate controls were selected, and retinal tissues were isolated after sacrifice. SDT buffer (4% SDS, 100 mM Tris–HCl, pH 7.6) was added to the sample. The lysate was homogenized, sonicated, and then boiled for 10 min. After centrifugation at 14,000 × *g* for 15 min, the supernatant was filtered through 0.22-μm filters. The proteins were quantified with a BCA protein assay kit (P0012, Beyotime). A total of 50–200 μg of protein/sample was reduced with 100 mM DTT for 5 min at 100 °C. The detergent, DTT, and low-molecular-weight components were removed using UA buffer (8 M urea, 150 mM Tris–HCl, pH 8.5) through repeated ultrafiltration (Sartorius, 30 KD). Iodoacetamide (100 mM IAA in UA buffer) was added to block reduced cysteine residues, and the samples were incubated for 30 min in darkness. The filters were washed three times with 100 μL of UA buffer and two times with 100 μL of 50 mM NH_4_HCO_3_ buffer. The protein suspensions were digested with 4 μg of trypsin (Promega) in 40 μL of 50 mM NH_4_HCO_3_ buffer overnight at 37 °C. The resulting peptides were collected as a filtrate and desalted on a C18 column.

#### Proteomic data analysis

Missing proteomic data were processed using the R package DEP (v1.16.0, https://bioconductor.org/packages/release/bioc/html/DEP.html) and then compared between groups by limma (v3.50.3, https://bioconductor.org/packages/release/bioc/html/limma.html) for determination of intergroup differences. The R package clusterProfiler was used for Kyoto Encyclopedia of Genes and Genomes (KEGG) pathway enrichment analysis. KEGG pathways with adjusted *P* values less than 0.05, as calculated by hypergeometric tests and the Benjamini–Hochberg method, were defined as significantly enriched pathways. The top 15 enriched pathways were selected for visualization.

### Transmission *electron* microscopy

Mice were sacrificed by cervical dislocation after anaesthesia, and the eye was quickly removed after the removal of the periocular tissue. The samples were briefly rinsed with 1 × PBS, and electron microscopy fixative (3% glutaraldehyde) was quickly injected into the eye to prefix the samples; the samples were then fixed with 1% osmium tetroxide. The tissue was then infiltrated sequentially with low to high acetone concentrations (30%, 50%, 70%, 80%, 90%, 95%, 100%) and dehydrated step by step. The samples were sequentially infiltrated with dehydrating agent and embedding agent at ratios of 3:1, 1:1, and 1:3, followed by embedding with embedding agent. Sections were sliced using a Leica (EM-UC7) ultrathin sectioning machine at 60–90-nm thickness, spread, and fixed to a copper mesh. The samples were stained by sequential immersion in uranyl acetate (10–15 min) and lead citrate (1–2 min). The copper mesh was first observed at 6000 × , and then, the target area was selected for image capture.

### Statistical analysis

All data are presented as the mean ± standard deviation (SD), and statistical analysis was performed using the GraphPad Prism version 8.0 software. Statistical significance was calculated using unpaired Student’s *t*-test or multiple Student’s *t*-tests. Values with *p*-value lower than 0.05 (*P* < 0.05) were considered statistically significant.

### Supplementary Information

**Additional file 1: Fig S1.** Single-cell RNA sequencing analysis of the mouse eye. **Fig S2.** Construction strategy and validation of retina-specific knockout of Pcyt1a mouse model. **Fig S3.** OCT images of two-month-old mice. **Fig S4.** H&E staining of retinas from 10-month-old mice. **Fig S5.** Immunofluorescence staining of retina in 2-month-old mice. **Fig S6.** Transmission electron microscopy of retinas from RKO and Ctl mice at 8-month of age. **Fig S7.** Detection of ferroptosis indicators in ARPE-19 cells without OA induction.

**Additional file 2: Table S1.** Protein Expression Results from 4D Label-Free Proteomic Analysis of Retinas from 2-Month-Old PCYT1A-RKO Mice. **Table S2.** qPCR-related primer sequences. **Table S3.** Retina Cell Clusters and Markers from Single-Cell RNA-Seq Data of Eye Tissue in 3-Month-Old Wild-Type Mice. **Table S4.** Key resources table.

**Additional file 3.** Uncropped blots. The raw blots corresponding with Figs. 5, 7 and Fig S2 are shown with molecular weight indicators.

## Data Availability

The dataset for the single-cell sequencing is available in the NCBI with accession PRJNA1103333 [61]. The source code for scRNAseq has been submitted to GitHub; the link is https://github.com/biolabcodes/data_analysis. The dataset supporting the proteomic results is included within the article (Additional file 2: Table S1). The mass spectrometry proteomics data have been deposited to the ProteomeXchange Consortium via the PRIDE partner repository with the dataset identifier PXD051537.

## Abbreviations

IRDs: Inherited retinal dystrophies
PC: Phosphatidylcholine
MG: Müller glia
INL: Inner nuclear layer
ERG: Electroretinogram
PL: Phospholipid
PE: Phosphatidylethanolamine
CCT: Phosphocholine cytidylyltransferase
LD: Lipid droplet
nLD: Nuclear lipid droplet
OCT: Optical Coherence Tomography
WB: Western blotting
FFAs: Free fatty acids
ROS: Reactive Oxygen Species Assay
FAO: Fatty Acid Oxidase Assay
α-KG: α-Ketoglutarate
ONL: Outer nuclear layer
OA: Oleic acid
PLIN2: Perilipin 2
OPL: Outer plexiform layer
IPL: Inner plexiform layer
DEPs: Differentially expressed proteins
FATP3: Fatty acid transporter 3
CPT1: Carnitine palmitoyltransferase1
DLD: Dihydrolipoamide dehydrogenase
GSH: Glutathione
xCT: Cystine transporter protein
GPX4: Ferroptosis inhibition protein
FTH1: Ferritin
Fer-1: Ferrostatin-1
CNS: Central nervous system
ELOVL2: Fatty acid elongase 2
TAG: Triacylglycerol
UMI: Unique molecular identifier
G6PD: Glucose-6-phosphate dehydrogenease
PKM1: Pyruvate kinase M1
HK1: Hexokinase-1
TSNE: T-distributed stochastic neighbour embedding
