# Author Correction: PCYT1A deficiency disturbs fatty acid metabolism and induces ferroptosis in the mouse retina

**DOI:** 10.1186/s12915-024-01954-6

**Published:** 2024-07-10

**Authors:** Kaifang Wang, Huijuan Xu, Rong Zou, Guangqun Zeng, Ye Yuan, Xianjun Zhu, Xiaohui Zhao, Jie Li, Lin Zhang

**Affiliations:** 1 The Sichuan Provincial Key Laboratory for Human Disease Gene Study, Center for Medical Genetics, School of Medicine, Sichuan Provincial People’s Hospital, University of Electronic Science and Technology of China, Chengdu, 610072 Sichuan China; 2 Qinghai Provincial Key Laboratory of Tibetan Medicine Research, Northwest Institute of Plateau Biology, Chinese Academy of Sciences, Xining, 810008 Qinghai China; 3 The People’s Hospital of Pengzhou, Chengdu, 611930 Sichuan China; 4 Medical Center Hospital of Qionglai City, Chengdu, 611530 Sichuan China; 5 Research Unit for Blindness Prevention of Chinese Academy of Medical Sciences (2019RU026), Sichuan Academy of Medical Sciences and Sichuan Provincial People’s Hospital, Chengdu, 610072 Sichuan China; 6 Department of Ophthalmology, School of Medicine, Sichuan Provincial People’s Hospital, University of Electronic Science and Technology of China, Chengdu, 610072 Sichuan China

**Author Correction: BMC Biology 22, 134 (2024)**

**https://doi.org/10.1186/s12915-024-01932-y**

The original article [1] mistakenly attributes co-author, Xiaohui Zhao to affiliation #3; Xioahui Zhao should instead be affiliated to affiliation #2.

## References

[1] Wang K (2024). PCYT1A deficiency disturbs fatty acid metabolism and induces ferroptosis in the mouse retina. BMC Biol.

