# Supplementary material for: PCYT1A deficiency disturbs fatty acid metabolism and induces ferroptosis in the mouse retina
Source: BMC Biol. 2024 Jun 10;22:134. doi: 10.1186/s12915-024-01932-y (PMC11165903; doi:10.1186/s12915-024-01932-y)
Supplement: Supplementary file 1 — Additional file 1: Fig S1. Single-cell RNA sequencing analysis of the mouse eye. Fig S2. Construction strategy and validation of retina-specific knockout of Pcyt1a mouse model. Fig S3. OCT images of two-month-old mice. Fig S4. H&E staining of retinas from 10-month-old mice. Fig S5. Immunofluorescence staining of retina in 2-month-old mice. Fig S6. Transmission electron microscopy of retinas from RKO and Ctl mice at 8-month of age. Fig S7. Detection of ferroptosis indicators in ARPE-19 cells without OA induction. [file 12915_2024_1932_MOESM1_ESM.docx]

**PCYT1A deficiency disturbs fatty acid metabolism**

**and induces ferroptosis in the mouse retina**

Kaifang Wang^1#^, Huijuan Xu^1,2#^, Rong Zou^1^, Guangqun Zeng^3^, YeYuan^4^, Xianjun Zhu^1,2,5^, Xiaohui Zhao^3^*, Jie Li^1,6^ *, Lin Zhang^1,2,5^*

^1^The Sichuan Provincial Key Laboratory for Human Disease Gene Study, Center for Medical Genetics, Sichuan Provincial People’s Hospital, School of Medicine, University of Electronic Science and Technology of China, Chengdu, Sichuan, 610072, China;

^2^ Qinghai Provincial Key Laboratory of Tibetan Medicine Research, Northwest Institute of Plateau Biology, Chinese Academy of Sciences Xining, Qinghai 810008, China;

^3^The people’s hospital of Pengzhou, Chengdu, Sichuan, 611930, China;

^4^ Medical Center Hospital of QiongLai City, Chengdu, Sichuan, 611530, China

^5^ Research Unit for Blindness Prevention of Chinese Academy of Medical Sciences (2019RU026), Sichuan Academy of Medical Sciences and Sichuan Provincial People’s Hospital, Chengdu, Sichuan, 610072 China.

^6^Department of Ophthalmology, Sichuan Provincial People's Hospital, School of Medicine, University of Electronic Science and Technology of China, Chengdu, Sichuan, 610072, China

^#^Authors contribute equally to this work

*Correspondence: Prof. Lin Zhang, [zhanglin202@uestc.edu.cn](mailto:zhanglin202@uestc.edu.cn) or Dr. Jie Li, [doctorjacklee@163.com](mailto:doctorjacklee@163.com) or Prof. Xiaohui Zhao, [xhzhao@nwipb.cas.cn](mailto:xhzhao@nwipb.cas.cn)


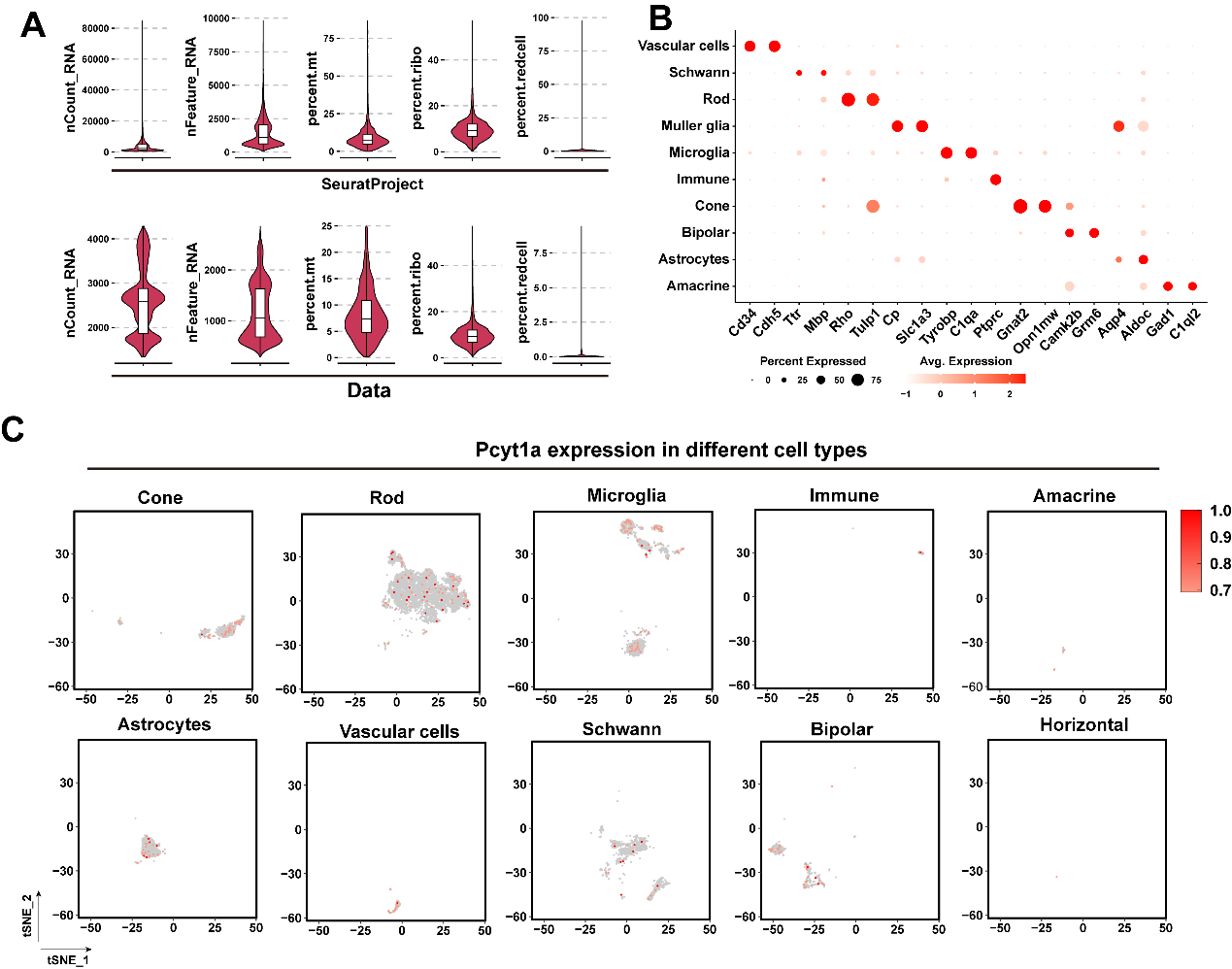


**Additional file 1: Figure S1. Single-cell RNA sequencing analysis of the mouse eye.** (A) Quality control of the single-cell RNA sequencing data involved filtering of low-quality cells. Cells were removed if they had <200 detected genes, >10% mitochondrial UMI counts, >10% hemoglobin gene UMI proportions, or were flagged as doublets by Scrublet. Pre- and post-filtering analysis is displayed in the upper and lower panels, respectively. (B) Dot plot of expression level and frequency among the 10 cell clusters. (C) t-SNE plot of *Pcyt1a* gene expression in different cell clusters.


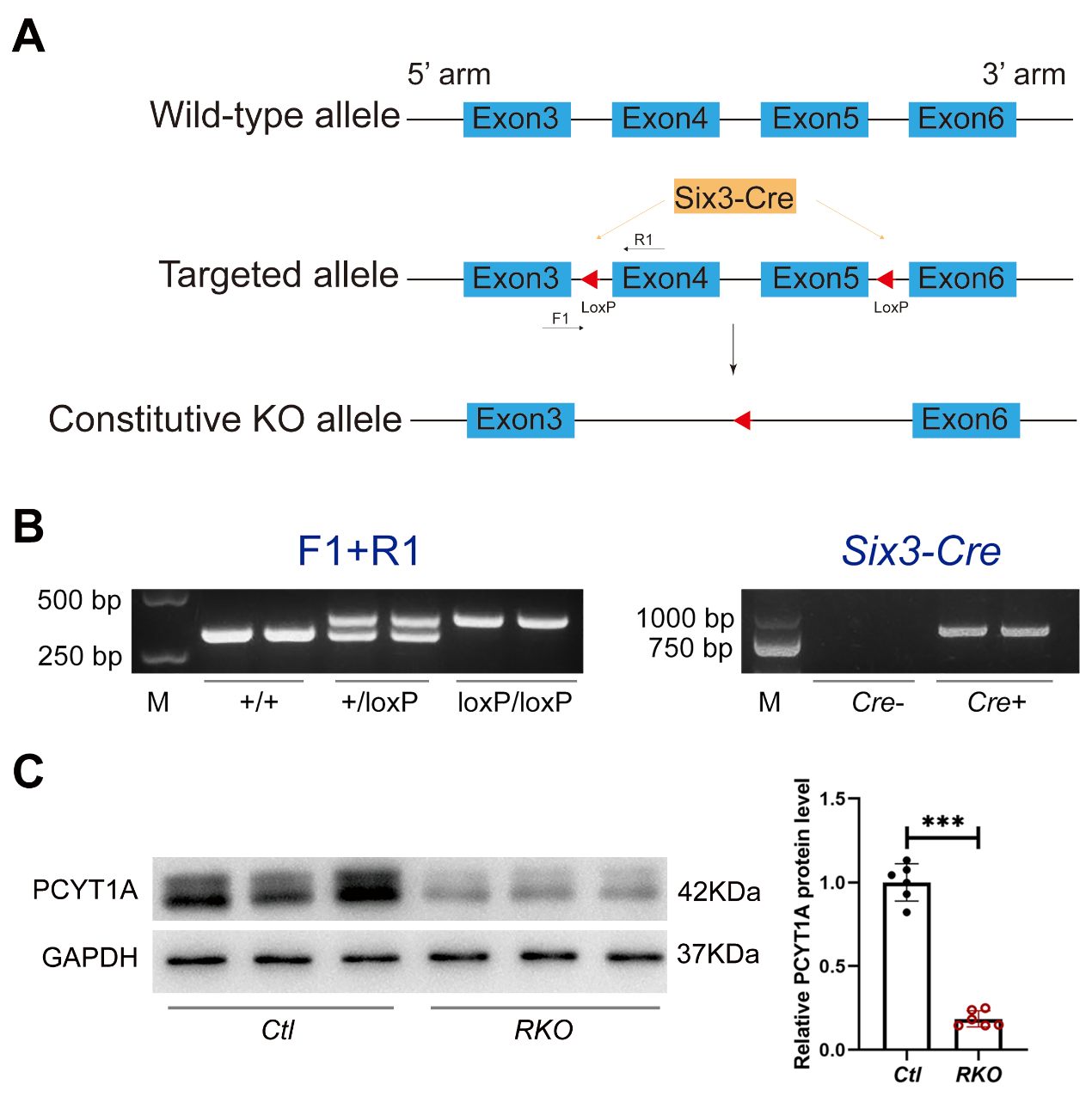


**Additional file 1: Figure S2. Construction strategy and validation of retina-specific knockout of *Pcyt1a* mouse model.** (A) Scheme showing the construction strategy for *Pcyt1a-RKO* mice. (B) Genotyping of RKO mice by PCR. (C) Immunoblotting experiments showed that the PCYT1A protein was reduced in RKO (n=6) mice compared to the Ctl (n=6) mice. ****P* < 0.001 by Student’s *t-*test. All data are shown as mean±SD.


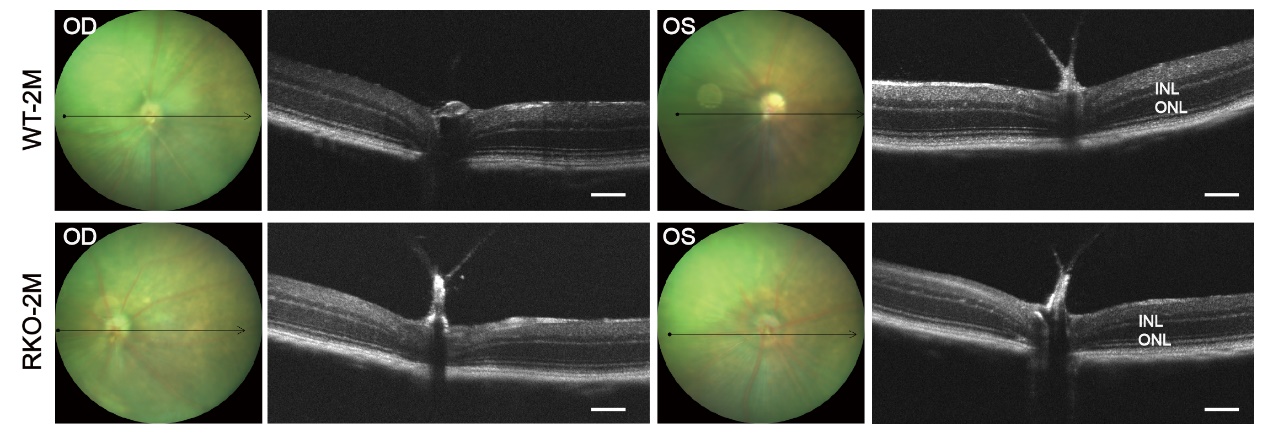


**Additional file 1: Figure S3.** Fundus images and OCT scanning photos on Ctl and RKO mice at 2-months of age. Scale bar 100μm


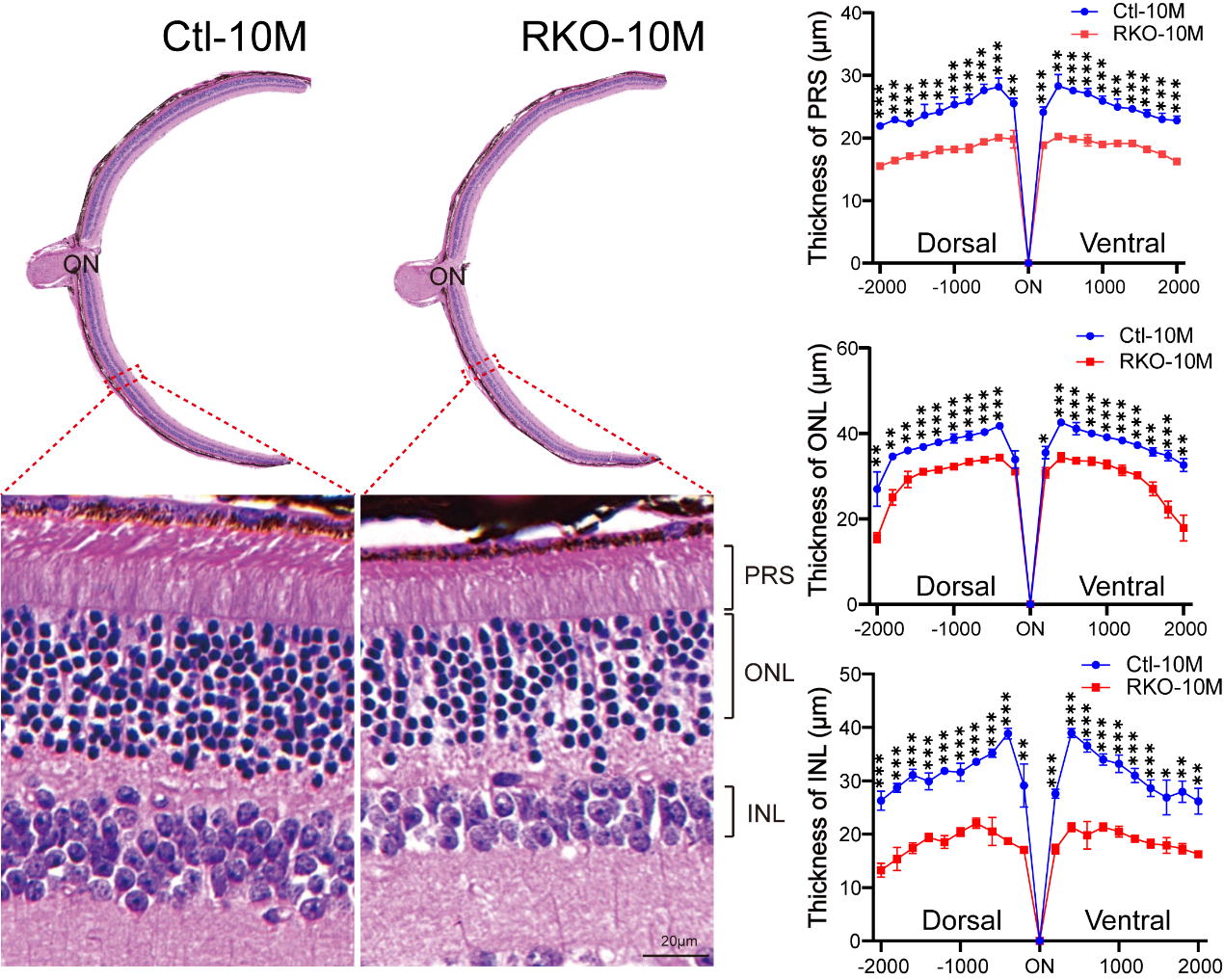


**Additional file 1: Figure S4. H&E staining of retinas from 10-month-old mice.** H&E staining on the retina from 10-month-old mice (left), and thickness quantification of the PRS, ONL and INL (right). Scale bar 20μm. PRS, photoreceptor segment; ONL, outer nuclear layer; INL, inner nuclear layer. The right panel shows the quantification of the thickness of the PRS, ONL and INL of 6 retinas from 3 Ctl mice and 6 retinas from 3 RKO mice at 10-month-old age. Multiple Student’s *t-*tests was used for statistical analysis. **P <* 0.05, ***P <* 0.01, ****P* < 0.001. All data are shown as mean±SD.


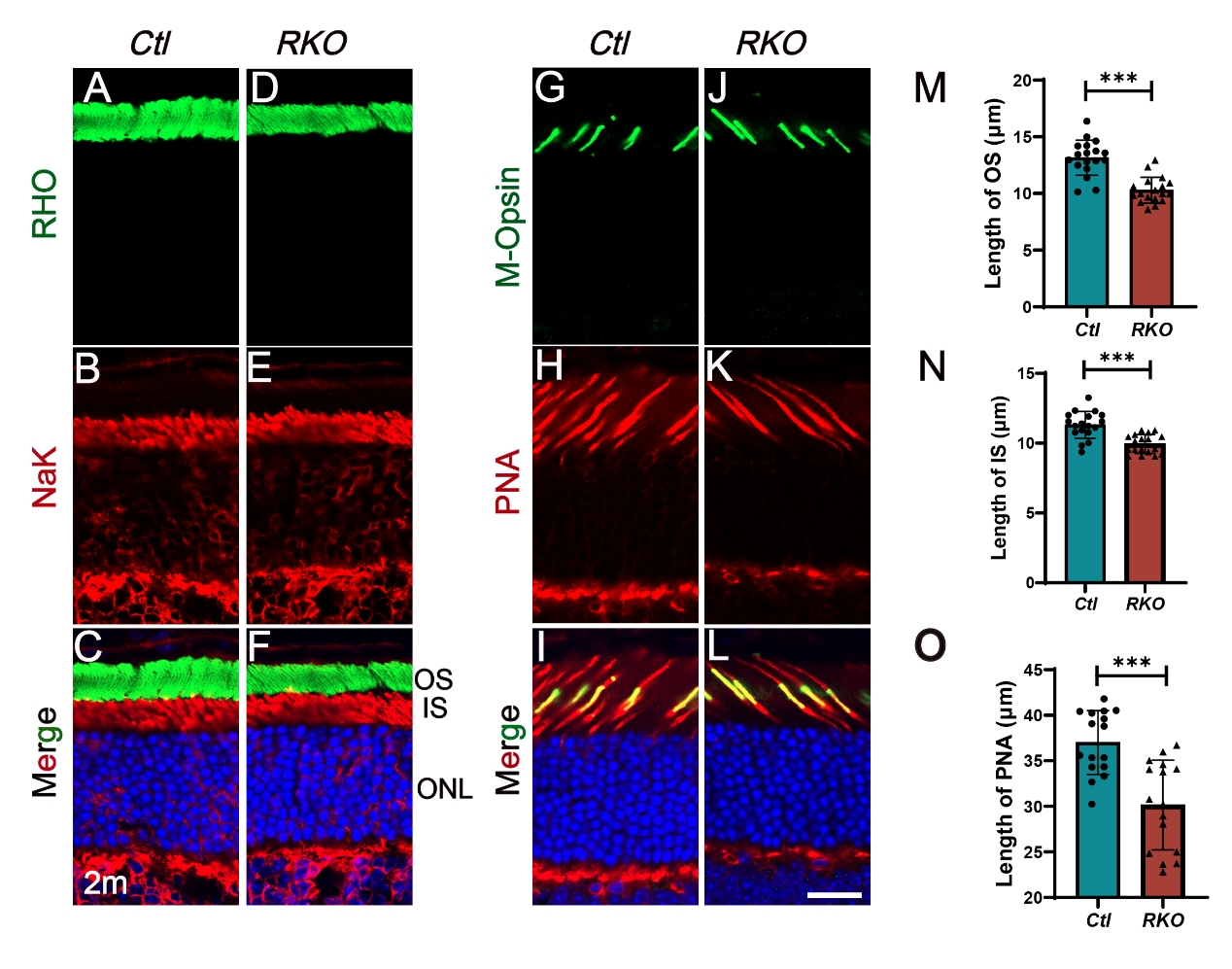


**Additional file 1: Figure S5. Immunofluorescence staining of retina in 2-month-old mice.**

(A-F) Immunofluorescence staining of retinal rod cells in 2-month-old mice. RHO in green, NaK in red, DAPI in blue. Scale bar, 20μm. OS, outer segment; IS, inner segment; ONL, outer nuclear layer. (G-L) Immunofluorescent staining of retinal cone cells from 2-month-old mice, M-opsin in green, PNA in red and DAPI in blue. Scale bar, 20μm. (M-O) Figures indicate the results of statistical analysis of the length of OS, IS and PNA (indicate the photoreceptor segment of cone cell). Statistical analysis of the lengths of the OS, IS and PNA (indicated by the PRS of the cone cell). The lengths of the OS, IS and PNA were measured using Zeiss software on the retinal sections at a distance of approximately 1000 μm from the optic center (both dorsal and ventral sides). Number of mice, n=3 for Ctl, and n=3 for RKO. Three sections from each mouse were randomly collected. Student’s *t-*tests was used for statistical analysis. ****P* < 0.001. All data are shown as mean±SD.


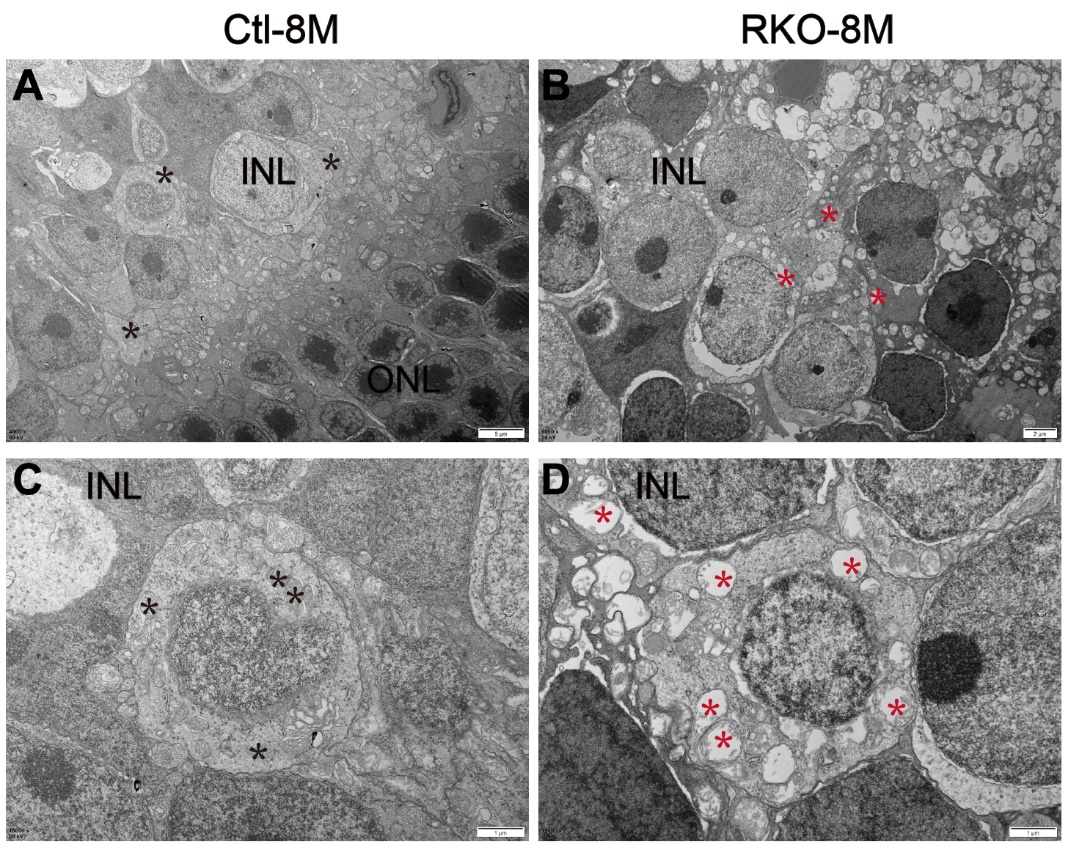


**Additional file 1: Figure S6. Transmission electron microscopy of retinas from RKO and Ctl mice at 8-month of age.** ONL outer nucleus player; INL, inner nucleus player; Asterisk in black indicate the mitochondria from INL of RKO retina; Asterisk in red indicate the mitochondria from INL of RKO retina; Scale bar, 2μm for A-B, and 1μm for C-D.


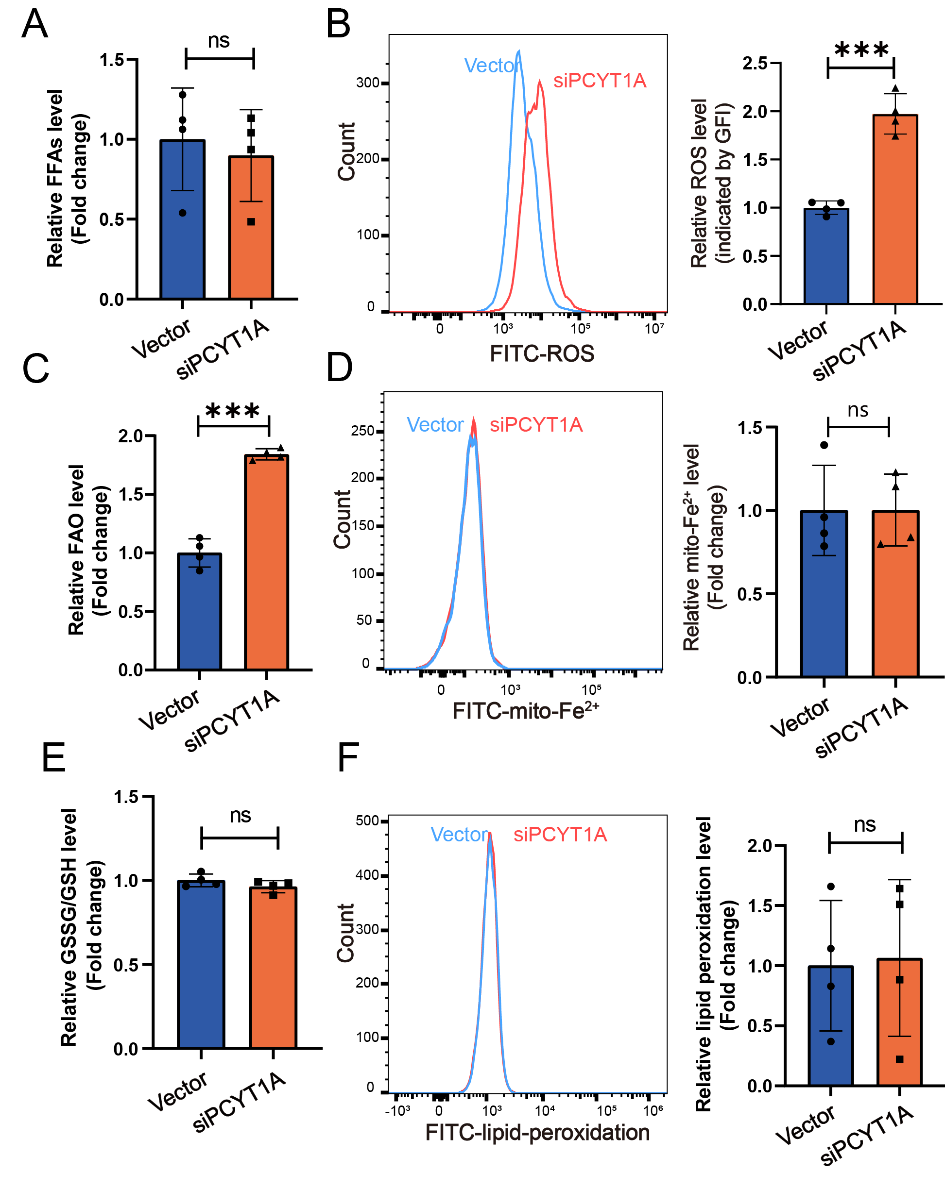


**Additional file 1: Figure S7. Detection of ferroptosis indicators in ARPE-19 cells without OA induction.** (A) There was no significant change in the levels of free fatty acids in siPCYT1A group (n=4) compared with Vector group (n=4). (B) ROS was increased in siPCYT1A (n=4) group compared with Vector group (n=4). Flow cytometry peaks are shown on the left, blue curve represents the Vector group and red curve represents the siPCYT1A group. Relative quantitative statistics are plotted on the right. (C) The siPCYT1A cells (n=4) exhibited higher FAO levels compared with Vector group (n=4). (D) Mitochondrial Fe^2+^ did not changed in siPCYT1A (n=4) group compared with Vector group (n=4). Flow cytometry peaks are shown on the left, blue curve represents the Vector group and red curve represents the siPCYT1A group. (E) GSSG/GSH levels did not changed in siPCYT1A cells (n=4) compared with Vector group (n=4). (F) Peroxidized lipids did not changed in siPCYT1A group (n=4) compared with Vector group (n=4). Flow cytometry peaks are shown on the left, blue curve represents the Vector group and red curve represents the siPCYT1A group. ****P*<0.001. ns, not significant. Student’s *t-*tests was used for statistical analysis. All data are shown as mean±SD.
