# Supplementary material for: PCYT1A deficiency disturbs fatty acid metabolism and induces ferroptosis in the mouse retina
Source: BMC Biol. 2024 Jun 10;22:134. doi: 10.1186/s12915-024-01932-y (PMC11165903; doi:10.1186/s12915-024-01932-y)
Supplement: Supplementary file 3 — Additional file 3. Uncropped blots. The raw blots corresponding with Figs. 5, 7 and Fig S2 are shown with molecular weight indicators. [file 12915_2024_1932_MOESM3_ESM.docx]

**PCYT1A deficiency disturbs fatty acid metabolism and induces ferroptosis in the mouse retina**

Kaifang Wang^1#^, Huijuan Xu^1,2#^, Rong Zou^1^, Guangqun Zeng^3^, YeYuan^4^, Xianjun Zhu^1,2,5^, Xiaohui Zhao^3^*, Jie Li^1,6^ *, Lin Zhang^1,2,5^*

^1^The Sichuan Provincial Key Laboratory for Human Disease Gene Study, Center for Medical Genetics, Sichuan Provincial People’s Hospital, School of Medicine, University of Electronic Science and Technology of China, Chengdu, Sichuan, 610072, China;

^2^ Qinghai Provincial Key Laboratory of Tibetan Medicine Research, Northwest Institute of Plateau Biology, Chinese Academy of Sciences Xining, Qinghai 810008, China;

^3^The people’s hospital of Pengzhou, Chengdu, Sichuan, 611930, China;

^4^ Medical Center Hospital of QiongLai City, Chengdu, Sichuan, 611530, China

^5^ Research Unit for Blindness Prevention of Chinese Academy of Medical Sciences (2019RU026), Sichuan Academy of Medical Sciences and Sichuan Provincial People’s Hospital, Chengdu, Sichuan, 610072 China.

^6^Department of Ophthalmology, Sichuan Provincial People's Hospital, School of Medicine, University of Electronic Science and Technology of China, Chengdu, Sichuan, 610072, China

^#^Authors contribute equally to this work

*Correspondence: Prof. Lin Zhang, [zhanglin202@uestc.edu.cn](mailto:zhanglin202@uestc.edu.cn) or Dr. Jie Li, [doctorjacklee@163.com](mailto:doctorjacklee@163.com) or Prof. Xiaohui Zhao, [xhzhao@nwipb.cas.cn](mailto:xhzhao@nwipb.cas.cn)

Uncropped western blotting figures

Figure5-uncropped:


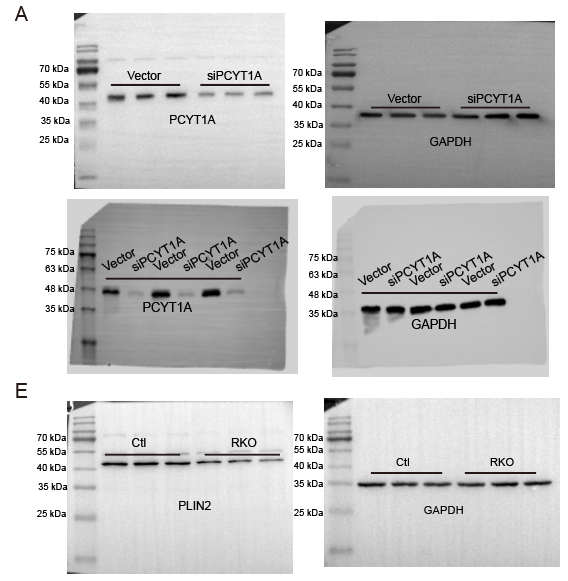


Figure7-uncropped:


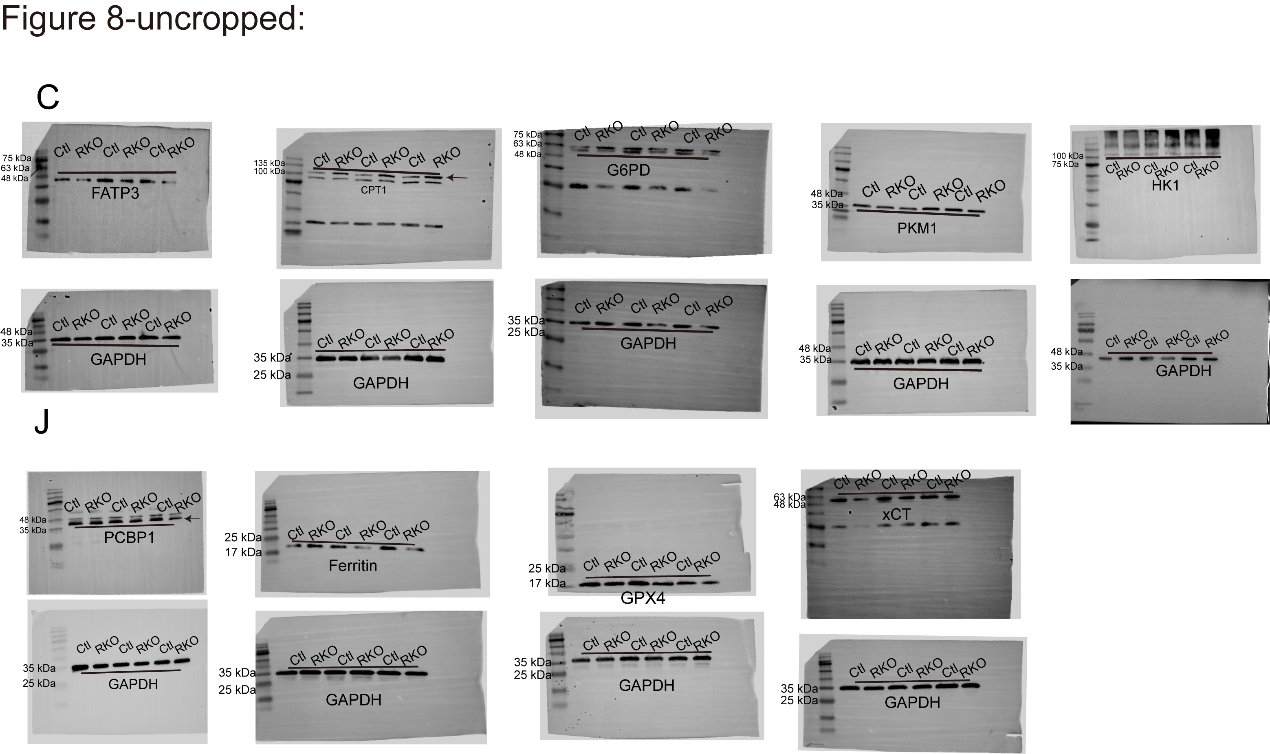


Additional File2: Fig. S2-uncropped:


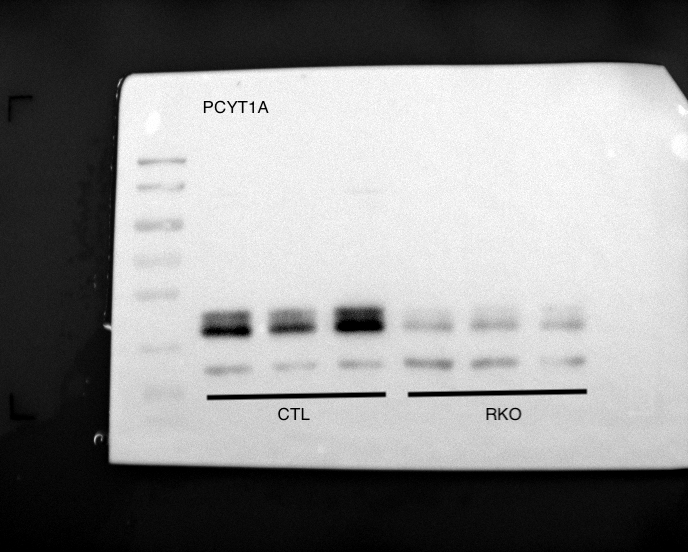

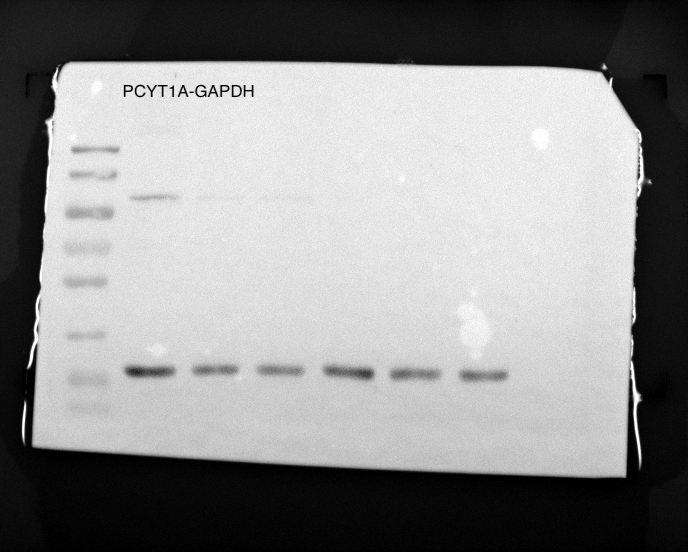


55 kDa

40 kDa

35 kDa

70 kDa

55 kDa

40 kDa

35 kDa

GAPDH


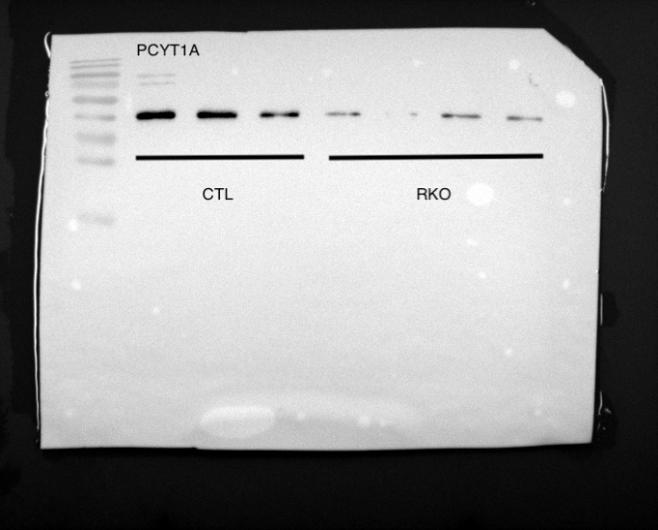

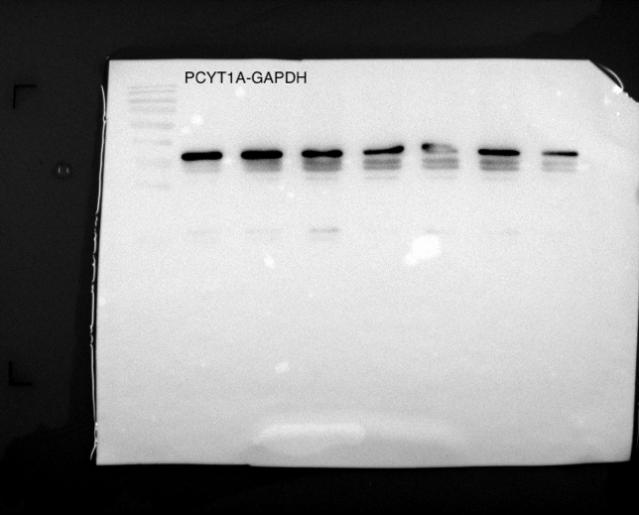


GAPDH

40 kDa

35 kDa

40 kDa

35 kDa

55 kDa
